# Disease‐associated gut microbiome and critical metabolomic alterations in patients with colorectal cancer

**DOI:** 10.1002/cam4.6194

**Published:** 2023-06-01

**Authors:** Hongze Zhang, Kai Jin, Kunlong Xiong, Wenwen Jing, Zhen Pang, Meng Feng, Xunjia Cheng

**Affiliations:** ^1^ Department of Medical Microbiology and Parasitology, School of Basic Medical Sciences Fudan University Shanghai China; ^2^ Department of Surgical Intensive Care Unit Huadong Hospital Affiliated to Fudan University Shanghai China; ^3^ Department of Respiratory and Critical Medicine Ningbo First Hospital Ningbo China; ^4^ Department of Hand Surgery, Huashan Hospital Fudan University Shanghai China

**Keywords:** 16S rDNA, colorectal cancer, *Fusobacterium nucleatum*, metagenomics, microbiome, taxonomic biomarker

## Abstract

**Background:**

Gut microbiota plays a significant role in the colorectal cancer (CRC) process. Ectopic colonization of multiple oral bacteria is reportedly associated with CRC pathogenesis and progression, but the details remain unclear.

**Methods:**

We enrolled a cohort of 50 CRC patients and 52 healthy controls from an East China population. Taxonomic and functional analysis of the fecal microbiota were performed using 16S rDNA (50 + 52 samples) and shotgun metagenomic sequencing (8 + 6 samples), respectively, with particular attention paid to gut‐colonized oral bacteria.

**Results and Conclusions:**

The results showed more detected bacterial species but lower species evenness within the samples from CRC patients. To determine the specific bacteria enriched in each group, we analyzed their possible protective, carcinogenic, or opportunistic roles in the CRC process. Among the ectopic oral bacteria, we observed a significant increase in the abundance of *Fusobacterium* and decreased abundance of *Prevotella* and *Ruminococcus* in the CRC group. Main differences in the functional composition of these two groups were related to energy metabolism and biosynthesis, especially the glycolytic pathway. Furthermore, we validated the colonization of *Fusobacterium nucleatum* subsp. *animalis* within CRC tissues and studied its impact on the host intestinal epithelium and tumor cells. With high selectivity for cancerous tissues, this subspecies promoted CRC cell proliferation and induced potential DNA damage.

## 1 INTRODUCTION

Representing approximately one in 10 cancer cases and deaths, colorectal cancer (CRC) ranked third in incidence and second in mortality worldwide in 2020.^1^, ^2^ Moreover, the incidence rates of CRC are not only particularly high in numerous developed regions, but have also been steadily rising in a series of Asian and South American transitioning countries.^3^ The cancer incidence and mortality profiles in China are changing from those of developing countries to those of developed ones.^4^ The rising incidence of CRC in China, especially in East China, mainly reflects changes in diet and lifestyle factors, such as an increased intake of animal‐source foods and decreased physical activity; the rapid aging of the population also amplifies the risk.^2^, ^5^ By 2020, CRC had replaced gastric cancer as the second most common cancer in China.^6^

The human colon harbors a highly sophisticated ecosystem of numerous bacteria, viruses, fungi, and protozoa, involving multiple essential functions of the host immune system and metabolism.^7^ Taxonomic and functional changes in the intestinal microbiota composition have been implicated in a variety of diseases throughout the human body, including CRC.^8^, ^9^, ^10^ Intestinal microorganisms can affect the occurrence and development of CRC in several ways,^11^ such as promoting sustained inflammation and weakening host immunity.

The oral cavity is an important gateway for microorganisms to enter the digestive tract. The average adult produces over 1000 mL of saliva each day, containing large amounts of oral bacteria.^12^, ^13^ The human actions of eating and swallowing give oral microorganisms a high opportunity to disseminate and colonize the gut.^14^ Oral bacteria may also enter the gastrointestinal tract via hematogenous routes during the frequent transient bacteremia caused by chewing, tooth brushing, or dental procedures.^15^, ^16^ These oral bacteria indirectly affect the composition of intestinal microbiota and interfere with intestinal homeostasis.^14^ In CRC patients, abundant bacteria of oral origin have been detected and validated within the intestinal microbiota, where they may play important roles in different stages of the disease.^17^, ^18^, ^19^

Gene amplicon sequencing is the primary technique for phylogenetic and taxonomic studies of complex microbiomes, among which 16S rRNA and 16S rDNA are the most commonly used target genes for microbial identification, also the gold standard for bacterial typing.^20^ However, 16S rRNA and 16S rDNA gene sequencing still have shortcomings in revealing the function of microbial communities. In this case, metagenomic analysis by the genome‐wide shotgun sequencing approach is becoming increasingly popular, which helps in associating function to phylogeny and taxonomy besides characterizing microbial community structure.^21^ Recently, modern next‐generation sequencing (NGS) has slowly replaced classical Sanger sequencing as a more precise and preferred tool for metagenomic shotgun sequencing.^20^ These microbial sequencing technologies have already made momentous contributions to the revelation of bacterial changes and human diseases.^22^

Using NGS technology, we examined the taxonomic and functional alterations of intestinal microbiota in healthy adults and CRC patients in East China, with a particular focus on bacteria of oral origin. We found the *Fusobacterium* strain significantly enriched in the gut of CRC patients and further verified its impact on host intestinal cells.

## 2 MATERIALS AND METHODS

### 2.1 Clinical samples and study population

Fresh stool samples from both newly diagnosed CRC patients before clinical treatment and healthy volunteers were collected in Huadong Hospital (Shanghai, China). In accordance with the *Helsinki Declaration*, the study protocol was subject to the approval of the Medical Ethics Committee of Huadong Hospital affiliated to Fudan University (permit AF16‐20170052). All participants were recruited with written informed consent prior to the study. Exclusion criteria for the two cohorts were as follows: age under 18, pregnancy, infection, chronic inflammation, mental disease, gastrointestinal surgery, previous history of other colonic diseases, use of antibiotics within the past 2 months, or refusal to participate after reading the informed consent form. All patients were diagnosed by colonoscopy and confirmed by pathological biopsy with complete clinical data. Stool samples were collected in sterile collection tubes in the hospital, snap‐frozen in liquid nitrogen, and stored at −80°C, finally treated within 1 month after collection.

### 2.2 Fecal DNA extraction

According to the manufacturer's instructions, DNA extraction was performed from 200 mg of fecal material with a QIAamp DNA stool Mini Kit (Qiagen). Genomic DNA integrity was measured through 1% agarose gel electrophoresis, while quality was estimated using a Nanodrop 2000 spectrophotometer (Nanodrop Technologies): concentration ≥20 ng/μL, volume ≥500 ng, A260/A280 = 1.8–2.0.

### 2.3 Amplification and sequencing of 16S rDNA

Specific forward (5′‐TCGTCGGCAGCGTCAGATGTGTATAAGAGACAGCCTACGG GNGGCWGCAG‐3′) and reverse (5′‐GTCTCGTGGGCTCGGAGATGTGTATAAGA GACAGGACTACHVGGGTATCTAATCC‐3′) primers targeting 16S rDNA gene V3–V4 variable regions were used for the amplification of the genomic DNA template. The amplicon was sequenced at Genesky Biotechnologies Inc., and three repeated experiments were set for each sample. Total DNA was purified and separated by use of Agencourt AMPure XP magnetic beads (Beckman Coulter). After an original library was formed by the addition of sample‐specific index sequences, it was quantified and pooled with the Invitrogen Qubit 3.0 Spectrophotometer (Thermo Fisher Scientific) and checked with the Agilent 2100 bioanalyzer (Agilent Technologies). The libraries were constructed by 2 × 250‐bp paired‐end sequencing on the Illumina MiSeq platform.

### 2.4 Shotgun metagenomic sequencing

Metagenomic sequencing was performed on stool samples of eight CRC patients and six healthy volunteers. Read pairs shorter than 35 bp in raw sequencing data were first filtered by Fastp 0.36; then, the adapter sequence was trimmed from the 3′ end of the reads with a quality threshold of 20 bp. To eliminate potential host DNA contamination in the samples, reads were aligned to the human genome by Bowtie 2.1.0. After discarding aligned reads, high‐quality reads were assembled into continuous sequences within scaffolds (scaftigs) using Megahit 1.2.9. The clean reads of each sample were mapped against the scaftigs by Bowtie, while the unmapped reads were continually assembled by SPAdes 3.13. During the assembly process, sequences under 500 bp were filtered from the dataset.

Subsequently, the scaftigs (≥500 bp) were used for open reading frame (ORF) prediction by Prodigal 2.60, among which predicted ORFs of more than 100 nt would be translated into amino acid sequences. Non‐redundant gene catalogs (unigenes) were established and clustered by CD‐HIT (identity 95%, coverage 90%) to obtain the longest representative sequences. Using Salmon 1.5.0, a bidirectional reasoning algorithm and deviation model were used to quantify gene abundance in each sample. The metagenomic sequencing was performed at Neo‐Biotechnology Co., Ltd.

### 2.5 Taxonomic and functional analysis

Based on the RDP and Silva databases for the taxonomic annotation, Mothur was used to cluster tags at a similarity level of 97% to obtain operational taxonomic units (OTU). BLASTP alignment was performed between the unigenes and sequences on the National Center for Biotechnology Information non‐redundant (NR) database with DIAMOND 0.8.20 (*e*‐value <1e‐5, score >60). The unigenes were aligned to the KEGG, Carbohydrate‐Active enZYmes (CAZy), and Uniprot databases to obtain functional annotations.^23^

### 2.6 Bacterial adherence determined by SPRi

Surface plasmon resonance imaging (SPRi) was implemented on an inverted optical microscope (IX83, Olympus) equipped with a 60× oil immersion objective. A superluminescent diode (SLD635B, Thorlabs) was used to excite surface plasmons from its collimated light, and plasmonic images were collected by a sCMOS camera (Photometrics Prime95B) at a frame rate of 100 frames/s with a view area of 102.4 × 102.4 μm^2^. Sensor chips were made with a BK‐7 glass coverslip coated with 2 nm chromium and 47 nm gold, to which the cells adhered and spread out. Flexible silicone microwells (350 μL; SARSTEDT) were mounted on top of the sensor surface through electrostatic interaction as reaction wells.

After the slow injection of *F. nucleatum* (5 × 10^4^ per mL of cell culture medium) under surface plasmonic resonance microscope, image sequences were recorded continuously for 700 s. The counting of single bacterial adherence to the cell membrane in the images was performed with a modified homemade automated particle counting algorithm as previously described.^24^

### 2.7 Cell migration assay

Scratch wound‐healing assays were conducted for examination of cell proliferation and migration. Caco‐2 or CCD 841 CoN cells were placed in 6‐well plates and co‐incubated with *F. nucleatum* (MOI = 20) or mFadA (5 μg/mL). After reaching 100% confluence, wound gaps were created by scratching the monolayer with the bottom of 200 μL pipette tips. Wound closure was visualized under an inverted phase‐contrast microscope, and ImageJ 2.1.0 software was used for the quantitative evaluation of wound healing area.

### 2.8 Quantitative real‐time RT‐PCR

The total RNA of Caco‐2 cells was purified with a RNeasy Plus Mini Kit (Qiagen) after the preincubation with *F. nucleatum* (MOI = 50) or mFadA protein (10 μg/mL). A PrimeScript first strand cDNA Synthesis Kit (TaKaRa) was used for reverse transcription. In accordance with manufacturer's protocols, the synthesized cDNA was used for qRT‐PCR in a final reaction volume of 20 μL on an ABI 7500 real‐time PCR system (Applied Biosystems). With a SYBR Premix Ex Taq (TaKaRa), reactions were performed in 96‐well plates under the following thermocycling conditions: 30 s at 95°C, 40 cycles of 5 s at 95°C, and 35 s at 60°C. Expressions of the human CHK2 gene were detected, while glyceraldehyde‐3‐phosphate dehydrogenase (GAPDH) was selected as the internal reference transcript. The primers are listed in Table S1. Gene expressions were analyzed using the 2^−ΔΔCt^ method.

### 2.9 Flow cytometry

Caco‐2 cells were preincubated with *F. nucleatum* (MOI = 50) or purified mFadA (10 μg/mL) for 24 h. After harvesting and washing, the cells were successively fixed with 4% paraformaldehyde fixative (Biosharp, #BL539A) and permeabilized with PBS containing 0.01% Triton X‐100. The cells were blocked with 5% bovine serum albumin in PBS and incubated with Alexa Fluor® 594 anti‐CHK2 antibody (BioLegend, #686603). Flow cytometry was performed with a BD FACSCalibur Flow Cytometer, while analysis was conducted using FlowJo v10.0.7 software.

### 2.10 Statistical analysis

R 3.6.3 was used for statistical calculations, and GraphPad Prism 8 (GraphPad Software, Version 8.3.0, USA) was used for statistical analysis. The differences between groups were examined by Student's *t*‐test or one‐way ANOVA (two‐tailed). Statistical significance was generally set at 0.05, while Bonferroni correction was applied on the significant level of multiple testing.

## 3 RESULTS

### 3.1 Clinical characteristics of the study participants

After the exclusion of off‐standard samples, we formed two study cohorts comprised of 50 CRC patients (tumor group) and 52 healthy individuals (control group). All study participants had lived in Shanghai for at least 2 years, reducing the effect of different diets and lifestyles on intestinal microbiota. The demographic characteristics of the two cohorts are shown in Table S2. The CRC patients were 65.32 ± 10.49 years of age, whereas the healthy volunteers were 54.13 ± 10.21 years of age. No significant differences in gender, race, or occurrence of diabetes were found between the two groups. The rates of obesity and hypertension were higher among CRC samples; differences also existed in alcohol and tobacco consumption. Clinicopathological characteristics of the CRC patients are shown in Table S3. As the most predominant pathological type, low differentiated adenocarcinoma accounted for 80% of the patients. The distribution of early‐stage disease (TNM I, II) and advanced‐stage disease (TNM III, IV) were found to be relatively equal; the primary origins of the tumor were also even.

### 3.2 Bacterial diversity and richness in fecal samples

**FIGURE 1:**
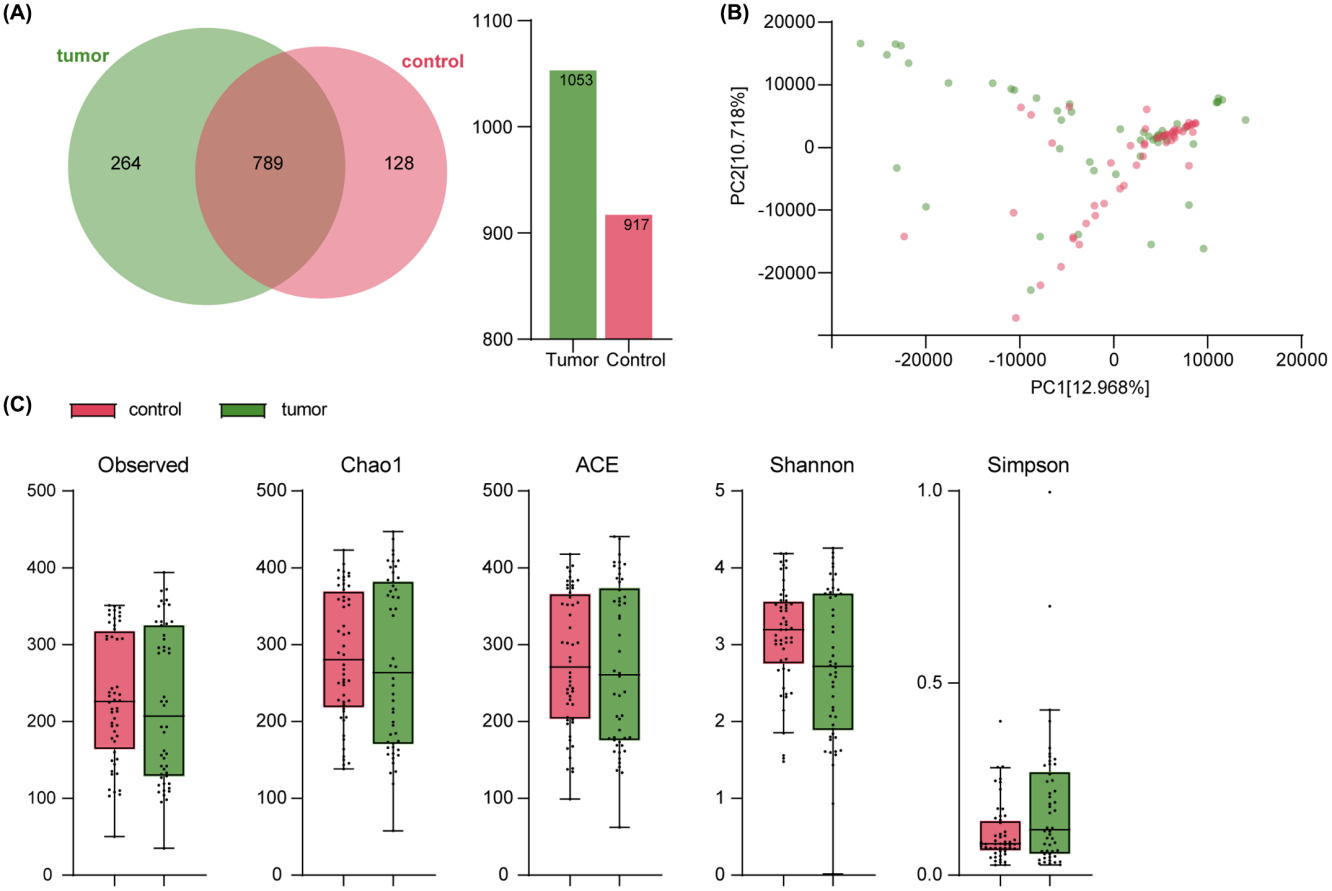
Alterations in bacterial richness and diversity in CRC patients. (A) The common and unique OTUs of the CRC and healthy groups. (B) Principal component analysis showed the compositional difference between the two groups: CRC (green) and healthy (red). (C) The differences in bacterial communities showed by various indices. The higher the observed OTUs/Chao 1/ACE value, the higher the species richness in the sample. A higher Shannon index or a lower Simpson index indicates higher species diversity. The figure used 16S rDNA sequencing data from 102 samples. CRC, colorectal cancer; OTU, operational taxonomic units.

A box plot of sequencing coverage (Figure S1A) and a rarefaction curve (Figure S1B) were used to evaluate the rationality of sequencing 102 samples. As the sequencing depth increased, the flatter curves indicated an adequate sequencing depth to cover almost all strains for subsequent analysis. The 16S rDNA sequencing showed 789 common OTUs between CRC and healthy groups (Figure 1A). A principal component analysis (PCA) plot showed the distinctions in β diversity of microbiota communities (Figure 1B). A significantly different trend of dispersion was observed in the two sample groups, suggesting a high variability of bacterial composition.

Community richness indices (Observed/Chao1/ACE) and community diversity indices (Shannon/Simpson) were further calculated to estimate the α diversity of bacterial components (Figure 1C). Though richness comparison revealed no difference between the two groups, the significant decrease in Shannon index and corresponding increase in Simpson index were found in CRC patients compared to healthy controls.

### 3.3 Taxonomy of gut microbiota in CRC patients and healthy controls

**FIGURE 2:**
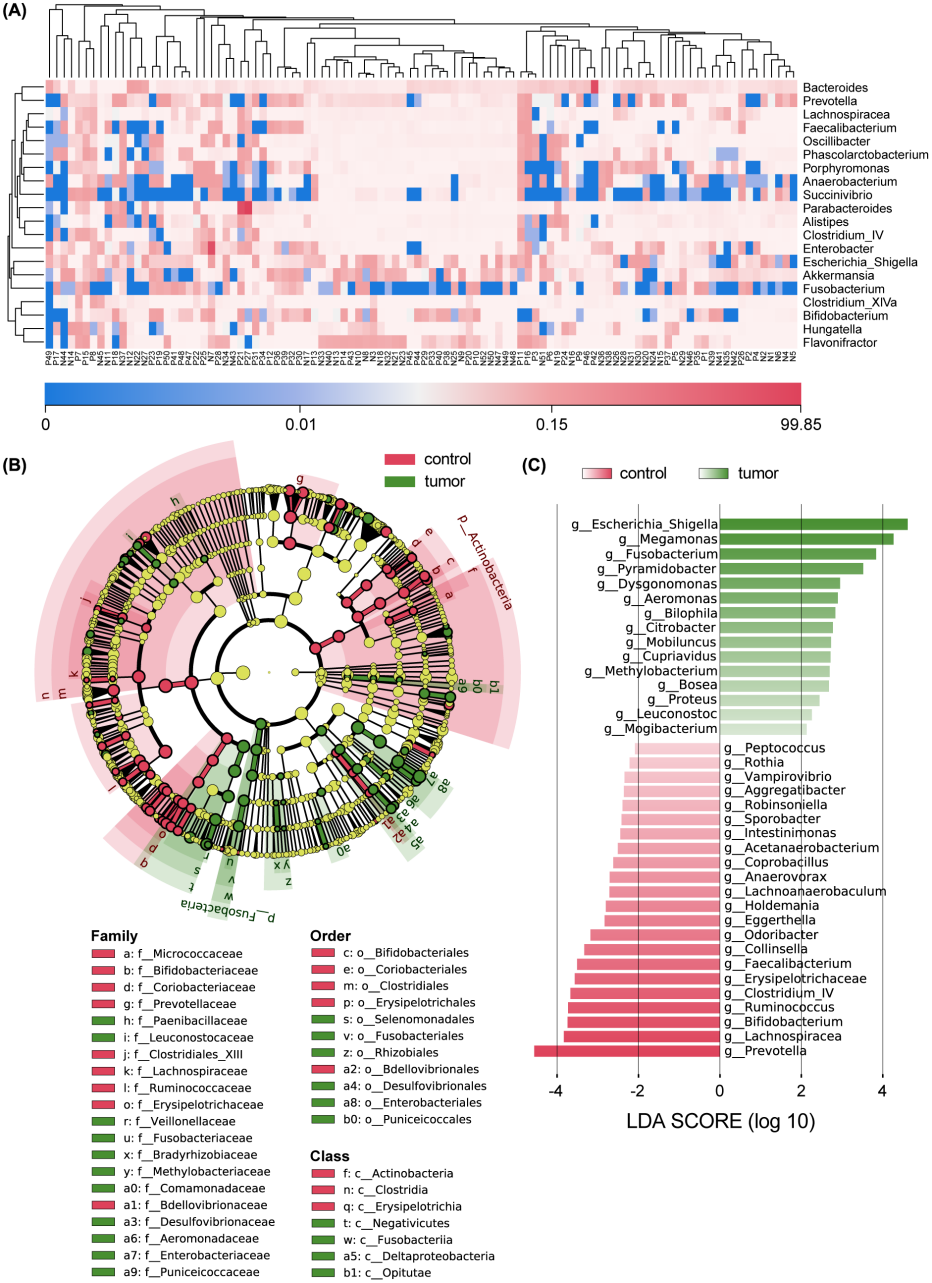
Differences in the relative abundance of intestinal bacteria at various levels. (A) Relative abundances of the top 20 bacterial genera in CRC and healthy samples. (B) LEfSe analysis of the relative abundance between the two groups. In the LEfSe cladogram, differences in relative abundance are represented as node sizes. Yellow nodes indicate bacteria with no significant difference, while green and red nodes indicate the taxa enriched in the CRC and healthy groups, respectively. The shading color shares the same meaning with the node color. (C) A histogram of different bacterial genera and LDA scores. LDA score >2.0 and *p* < 0.05 were set as the criteria for significant discrimination. The figure used 16S rDNA sequencing data from 102 samples. CRC, colorectal cancer; LDA, linear discriminant analysis.

The analysis of fecal bacteria distribution at the phylum level indicated the predominance of *Bacteroidetes*, *Firmicutes*, and *Proteobacteria* in the majority of samples (Figure S2). Other phyla such as *Verrucomicrobia* and *Fusobacteria* were also relatively abundant in many samples of each group. At the genus level, *Bacteroides*, *Prevotella*, and *Lachnospiracea* were the most abundant, while the abundance of some bacteria, such as *Succinivibrio* and *Fusobacterium*, varied greatly among different samples (Figure 2A).

From class level to family level, the LEfSe cladogram showed all the significantly different bacteria between the two groups (Figure 2B). The differential bacteria of the CRC group were mainly in phyla *Fusobacteria* and *Proteobacteria*, and the differential bacteria of the healthy group were mainly in phyla *Actinobacteria* and *Firmicutes*. The linear discriminant analysis (LDA) histogram showed that CRC patients were characterized by a higher abundance of *Escherichia‐Shigella*, *Megamonas*, *Fusobacterium*, *Pyramidobacter*, and *Dysgonomonas*, while healthy controls exhibited higher enrichments with *Prevotella*, *Lachnospiracea*, *Bifidobacterium*, *Ruminococcus*, and *Clostridium* IV (Figure 2C).

### 3.4 Different abundances of dominant oral bacteria in the gut

At the genus level, we found a high relative abundance of some oral bacteria, such as *Prevotella* and *Fusobacterium*, in fecal samples (Figure 2A). The significantly different enrichment of several oral bacteria between CRC and healthy groups was also shown in Figure 2B,C. The relative abundance of *Fusobacteriaceae* was higher in CRC patients, while the relative abundance of *Lachnospiraceae*, *Ruminococcaceae*, and *Prevotellaceae* was higher in healthy controls.

**FIGURE 3:**
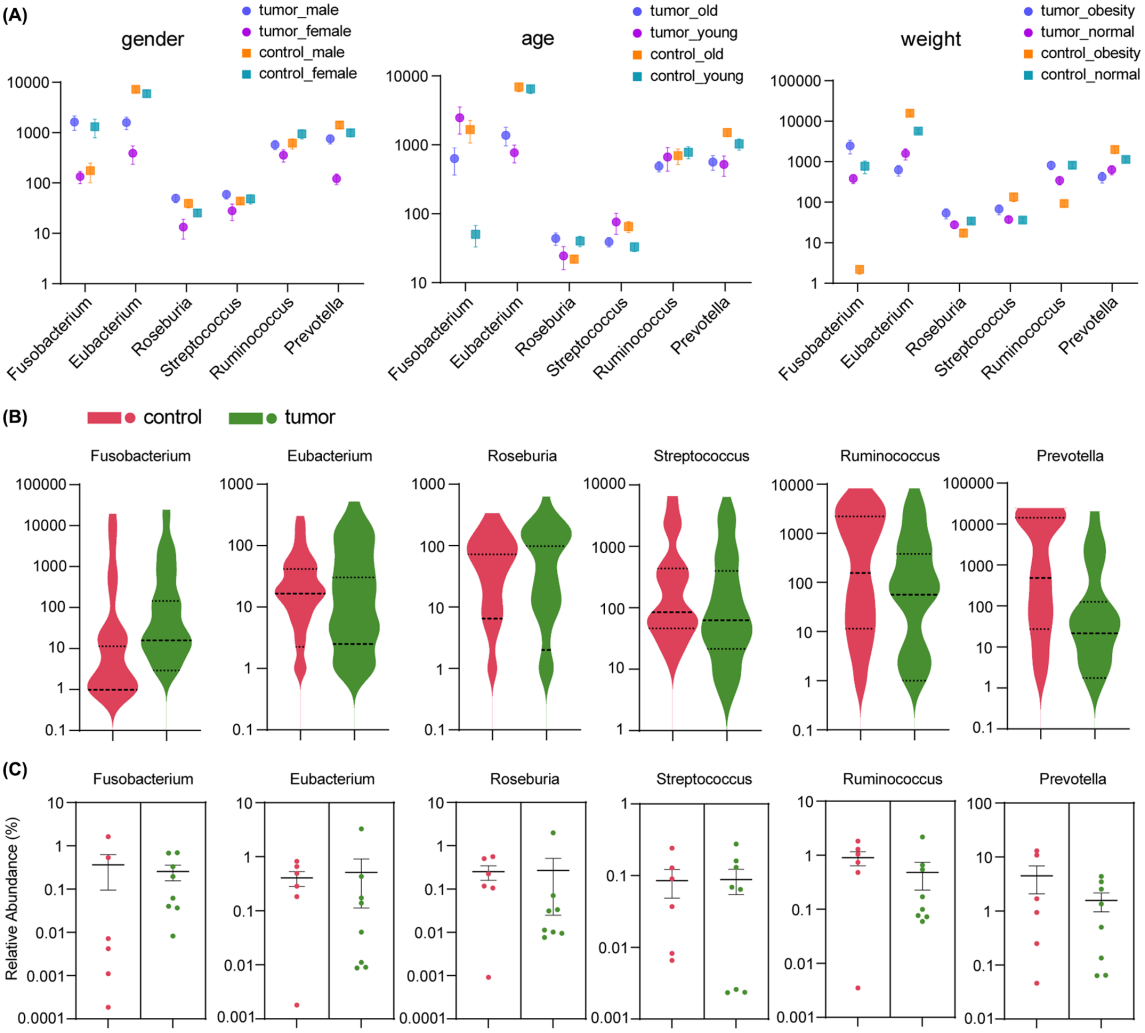
Intestinal abundance of oral bacteria in different CRC and healthy groups. (A) Intestinal abundance of the six oral bacterial genera in each group under the classification standards of sex, age, or weight. (B) Differences in the intestinal abundance of the six oral bacterial genera between the CRC and healthy groups. (C) Relative abundance differences of the six oral bacterial genera in the intestinal tract between two selected sample groups. Eight CRC patients (P3, P6, P11, P13, P16, P24, P42, and P46) and six healthy volunteers (N16, N17, N19, N36, N38, and N51) with overall similarity were selected to minimize the differences in intestinal abundance of oral bacteria between the two groups. CRC, colorectal cancer.

We focused on six important oral bacterial genera with high abundance in fecal samples.^14^, ^25^, ^26^ Under the further classification criteria of sex, age, or weight, the abundance of six oral bacteria genera exhibited no significant difference among several CRC and healthy groups (Figure 3A). However, based on this analysis of 102 samples, the abundance of *Fusobacterium* in the Control Young or Control Obesity group was significantly lower than that in other groups. To reduce the impact of excessively different sample characteristics on relative abundance and functional prediction of the ectopic oral bacteria, eight CRC patients (P3, P6, P11, P13, P16, P24, P42, and P46) and six healthy controls (N16, N17, N19, N36, N38, and N51) were selected for subsequent analysis according to the similarity of their clinical features and the taxonomic composition of their intestinal microbiota. After selection, no significant differences in age, weight, hypertension, or alcohol/tobacco consumption existed (Table S4). Finally, as shown in Figure 3B,C, the disparity in intestinal abundance of oral bacteria between the two selected sample groups was greatly minimized.

Shotgun metagenomic sequencing was performed on the 14 fecal samples. In all, 3005 common OTUs were found between CRC and healthy groups (Figure S3A). In comparison with the corresponding results from 16S rDNA sequencing (Figure 1A), the proportions of respective and common OTUs between the two groups were highly identical, indicating the consistency of the two sequencing methods. The PCA analysis exhibited a more concentrated distribution of the 14 samples (Figure S3B), which manifested an effective reduction in the distinctions in microbial community β diversity compared with the results from 102 samples (Figure 1B). *Bacteroidetes*, *Firmicutes*, and *Proteobacteria* were still the three predominant phyla in the 14 samples (Figure S3C), but the composition of intestinal microbiota in each sample showed higher similarity (compared to Figure S2). At the genus level, the top 20 sources of differences between the two groups were calculated by Random Forest analysis (Figure S3D). The abundances of several oral bacterial genera, such as *Fusobacterium* and *Prevotella*, were initially significantly different between the CRC and healthy groups (Figure 2C), but now more similar in the 14 selected samples. No oral bacteria were among the top 20 sources of differences.

### 3.5 Functional prediction of the global microbiome and dominant oral bacteria

**FIGURE 4:**
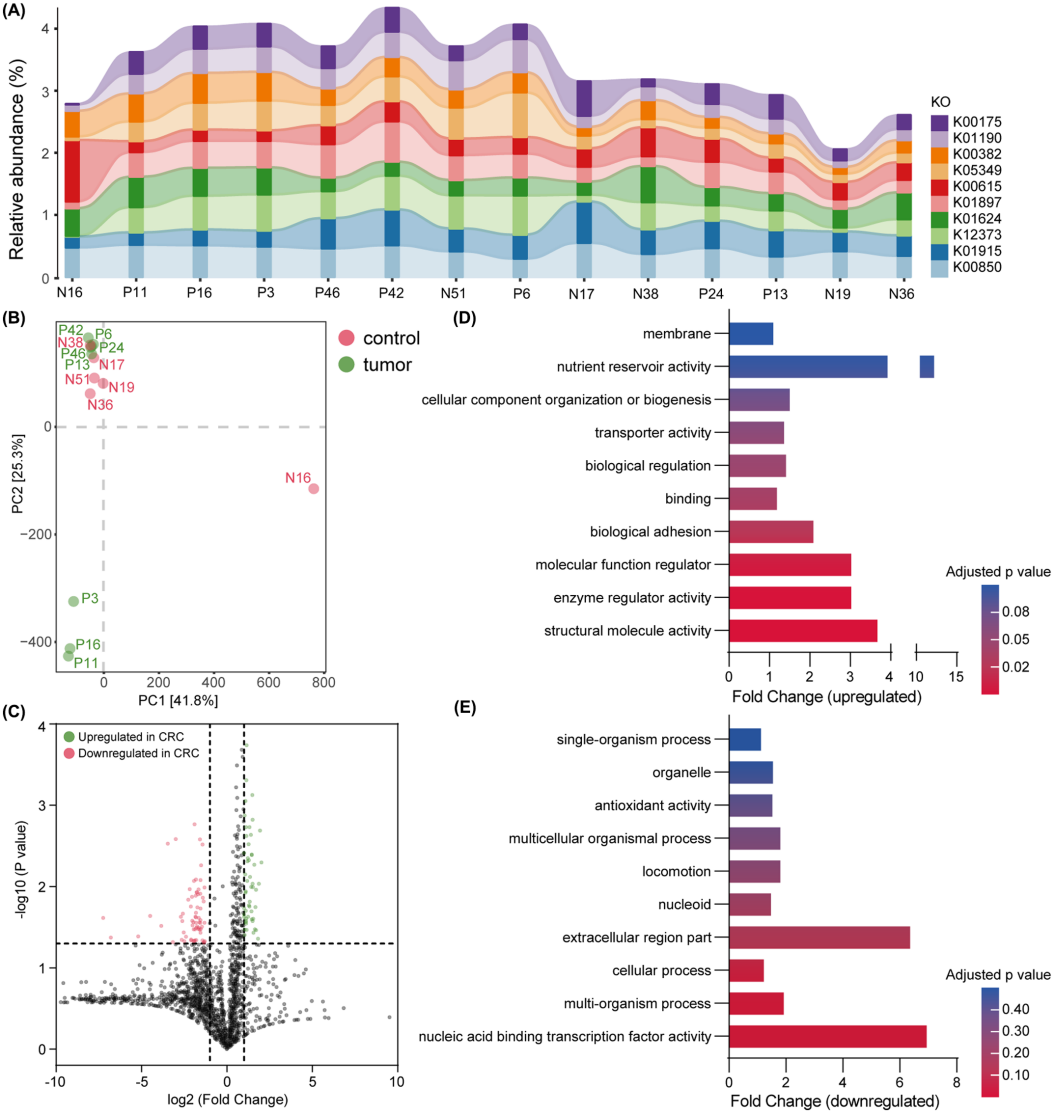
Functional prediction of intestinal bacteria in eight CRC patients and six healthy volunteers. (A) The relative abundance of the 10 most expressed orthologous genes in each sample. (B) Principal component analysis of intestinal bacterial KEGG pathways: healthy (red) and CRC (green). (C) A Volcano plot of gene expression fold changes in the CRC group versus the healthy group: upregulation (green) and downregulation (red). (D) Top 10 upregulated functions in the CRC group (Level 2) by GO enrichment analysis. (E) Top 10 downregulated functions in the CRC group (Level 2) by GO enrichment analysis. Shotgun metagenomic sequencing data were used in the figure. CRC, colorectal cancer.

Using DIAMOND software, the functional prediction of metagenome analysis was implemented via comparison with the KEGG, Uniprot, and CAZy databases. Of all predicted genes annotated in the KEGG database, 74,909 were further annotated to 2903 KEGG Orthology Groups. Relative abundances of the 10 most expressed orthologous genes in the samples are shown in Figure 4A and Table S5, mainly involving the metabolism of carbohydrate, amino acid, and fatty acid. Similar to the PCA analysis of bacterial composition (Figure S3B), no significant difference was observed at the pathway level between CRC and healthy groups (Figure 4B). In the CRC patient group, the expression levels of 123 genes were significantly changed compared with those in healthy controls, among which 54 (2.12% of total) were upregulated and 69 (2.71% of total) were downregulated (Log_2_FC < −1 or Log_2_FC > 1, *p* < 0.05 in Student's *t*‐test) (Figure 4C).

GO enrichment analysis was implemented to determine the biological process (BP), cell component (CC), and molecular function (MF) of differentially expressed genes. Divided into upregulated and downregulated changes, the top 10 significantly altered functions (Student's *t*‐test) in the CRC group are shown in Figure 4D,E, respectively. The significantly upregulated functions in CRC patients, such as metabolism, adhesion, and binding, were concentrated in the components of BP and MF, whereas the significantly downregulated functions were mainly related to nucleic acid transcription. After alignment to the CAZy database using HMMER3 software, the CRC group exhibited a higher global activity of carbohydrate‐related enzymes compared with the healthy group (Figure S4).

We further analyzed the functional differences of the ectopic oral bacteria. After removing the factor of different bacterial abundances between the two groups, *Fusobacterium* (FC = 3.48) and *Streptococcus* (FC = 2.41) exhibited higher levels of global gene expression in CRC patients, whereas *Prevotella* (FC = 16.45) and *Ruminococcus* (FC = 12.17) exhibited higher levels of global gene expression in healthy controls (Figure S5A). The gene expression of *Eubacterium* (FC = 1.01) or *Roseburia* (FC = 1.56) showed little difference between the two sample groups. Ranked by the relative abundance of gene expression, the top 100 orthologous genes exhibited different expression characteristics among the oral bacteria in the CRC group (Figure S5B). In general, the gene expression of *Fusobacterium* was significantly upregulated in CRC patients, whereas the most discriminative downregulation of gene expression was found in *Ruminococcus* and *Prevotella*. Aligned with the CAZy database, *Fusobacterium* exhibited a significant upregulation of carbohydrate‐related metabolism in the CRC group, and *Prevotella* had the most significant downregulation (Figure S5C).

### 3.6 *Fusobacterium* in fecal and intestinal tissue specimens

**FIGURE 5:**
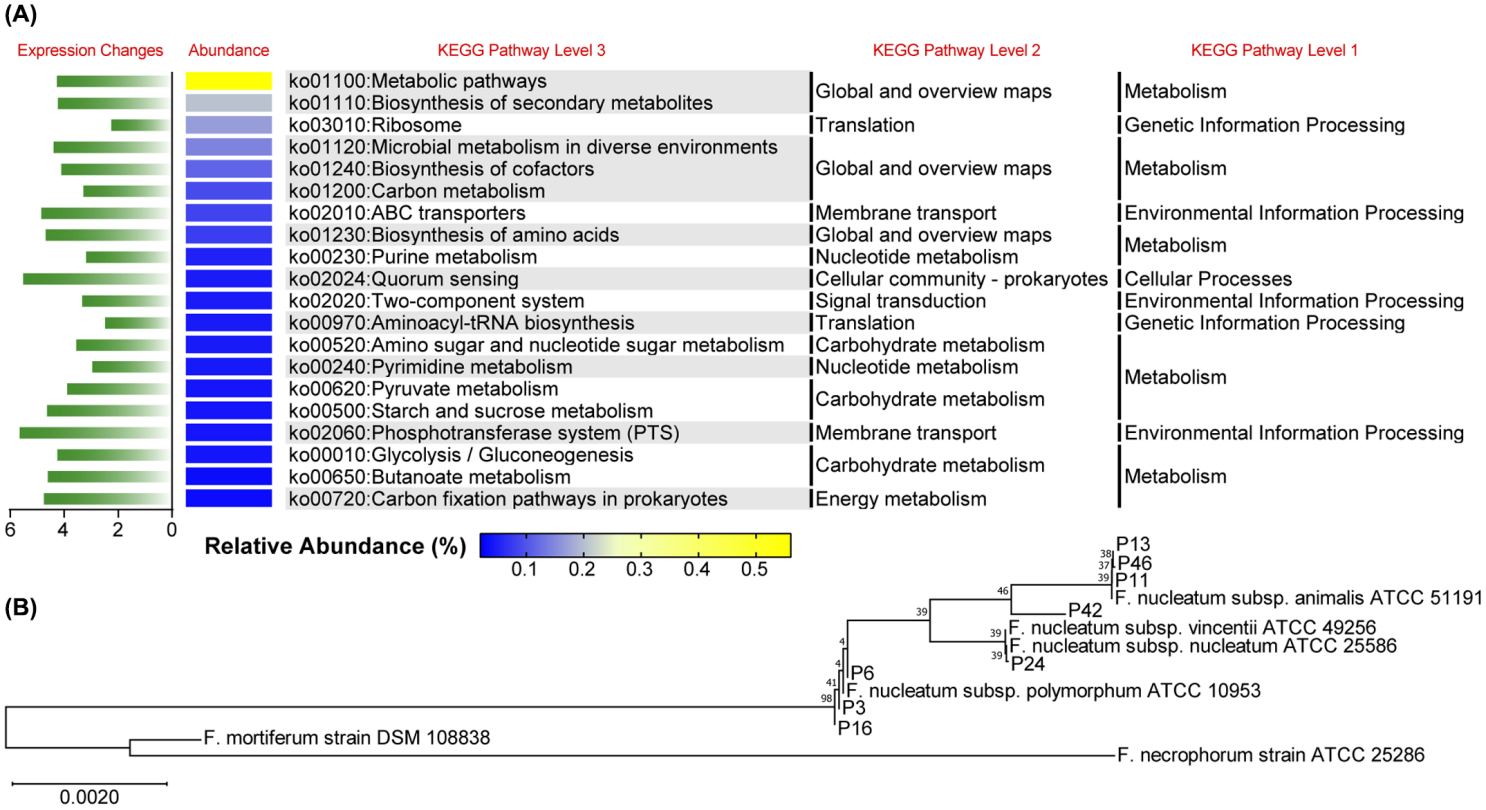
Functional prediction and colonization validation of *Fusobacterium*. (A) Targeting the genus *Fusobacterium*, fold changes in the gene expression of the 20 most abundant pathways between the CRC and healthy sample groups. The comparison was conducted using the KEGG database, and the green color represents higher expression in CRC samples. (B) Genetic relationships among the *Fusobacteria* in eight patient samples (colonic biopsy specimens) and species/subspecies of the genus. Assessed by a bootstrap analysis of 1000 replicates using MEGA version 11.0.13, a neighbor‐joining phylogenetic tree was constructed to validate the *Fusobacterium* colonization in intestinal tissues. Bar indicates 0.0020 changes per nucleotide position. Shotgun metagenomic sequencing data was used in the figure. CRC, colorectal cancer.

As the most enriched oral bacteria in fecal samples from CRC patients, the upregulation of functional gene expression in *Fusobacterium* was also the most significant. Among the 100 most abundant orthologous genes, the expression of 16 ones was significantly upregulated (Log_10_FC >1), mainly involving bacterial metabolism and biosynthesis (Figure S5B; Table S6). Ranked by the relative abundance of pathway expressions, the expression levels of *Fusobacterium*'s top 20 pathways were all increased in the CRC group (Figure 5A). The abundance of ko02024 (quorum sensing, FC = 5.52) and ko02060 (phosphotransferase system, FC = 5.65) were altered most significantly. Based on the metagenomic result from fecal samples, formalin‐fixed and paraffin‐embedded colonic biopsy specimens of the eight CRC patients were obtained to verify the colonization of *Fusobacterium* in tumor tissues. Compared with several of the most common clinically infected *Fusobacterium* species, bacteria from the eight specimens exhibited the closest genetic relationship to *F. nucleatum*, especially to the subsp. *animalis* (Figure 5B).

### 3.7 Selectivity of *Fusobacterium nucleatum* to human intestinal cells

**FIGURE 6:**
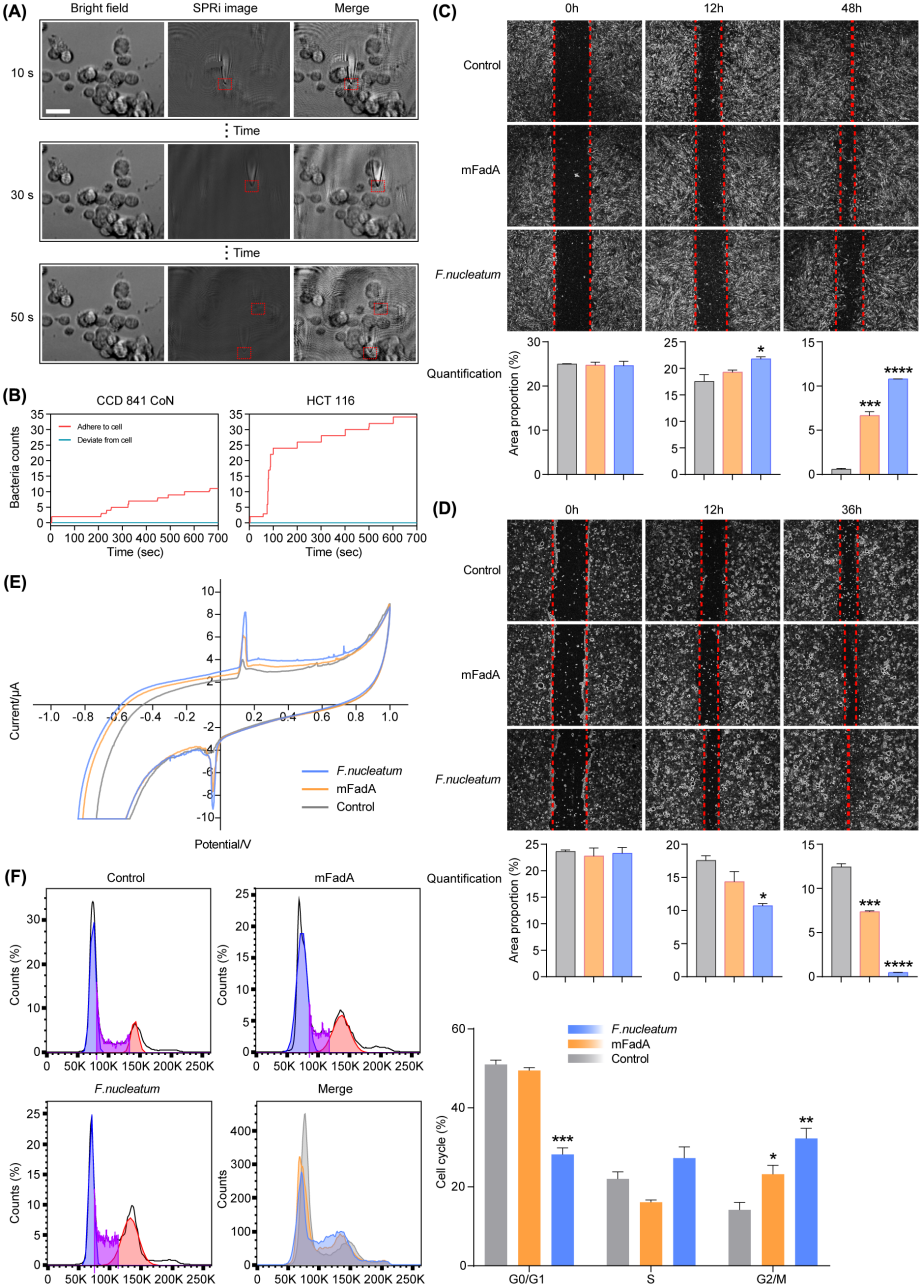
*Fusobacterium nucleatum* tends to adhere to colorectal cancer cells and promote their proliferation. (A) A schematic diagram of bacterial adherence detection by SPRi. HCT 116 cells were taken as the example. Scale bar: 20 μm. (B) The bacterial adherence to CCD 841 CoN and HCT 116 cells at the single‐bacteria‐cell level. The adherence and deviation of *F. nucleatum* are shown, respectively. (C) The electrochemical response of Caco‐2 cells after in situ lysis. The cells were preincubated with *F. nucleatum* or mFadA protein for 24 h. Scan rate: 0.1 V/s. Temperature: 37 ± 0.5°C. (D) Wound‐healing assay and the quantification of wound area in CCD 841 CoN cells after treatment with *F. nucleatum* or mFadA. (E) Wound‐healing assay and the quantification of wound area in Caco‐2 cells after treatment with *F. nucleatum* or mFadA. (F) Cell cycle analysis of Caco‐2 cells detected by flow cytometry. The cells underwent a 24 h preincubation with *F. nucleatum* or mFadA. Data are expressed as mean with SD. *p < 0.05; **p < 0.01; ***p < 0.001; ****p < 0.0001. The *F. nucleatum*, mFadA, and control groups are respectively marked in blue, yellow, and gray.

According to the sequencing results of intestinal tissue specimens, *F. nucleatum* subsp. *animalis* was selected for functional validation on host cells. To directly investigate the adhesion and invasion of *F. nucleatum* in normal and malignant colorectal cells, plasma imaging time‐resolved counting experiments were performed at the single‐bacteria‐cell level on CCD 841 CoN and HCT 116 cells. The schematic diagram of bacterial adherence to the cell surface at different time points was shown in Figure 6A. After the algorithm process for observing bacterial attachment, the SPRi image was superimposed on the bright field image to form the merged image, in which the adhesion of a single bacterium to the cell surface could be easily confirmed. In the 700 s continuous signal detection, the average number of *F. nucleatum* attached to a unit area of CCD 841 CoN cells was 11, while the average number on a unit area of HCT 116 cells was 34 (Figure 6B). *F. nucleatum* exhibited obvious selectivity for CRC cells.

To further determine the effect of *F. nucleatum* on normal and malignant cells, wound gaps were generated to examine cell proliferation and migration. During the co‐incubation with *F. nucleatum* or mature FadA (mFadA) protein, the wound healing of CCD 841 CoN cells was retarded (Figure 6C), while that of Caco‐2 cells was accelerated (Figure 6D). On the whole, the wound‐healing rate of malignant cells was significantly higher than that of the normal cells. Considering the significantly higher selectivity and proliferation‐promoting effect, the CRC cell line (Caco‐2) was selected for further investigation.

### 3.8 Regulation of colorectal cancer cells by *F. nucleatum* and mFadA protein

The effect of *F. nucleatum* or mFadA on the cyclic voltammetry response of Caco‐2 cells is shown in Figure 6E. The augment in peak current was likely related to the increased viability and proliferation of the cells.^27^ A cell cycle analysis of Caco‐2 cells again verified the promoting effect of *F. nucleatum* and mFadA on cell proliferation (Figure 6F).

**FIGURE 7:**
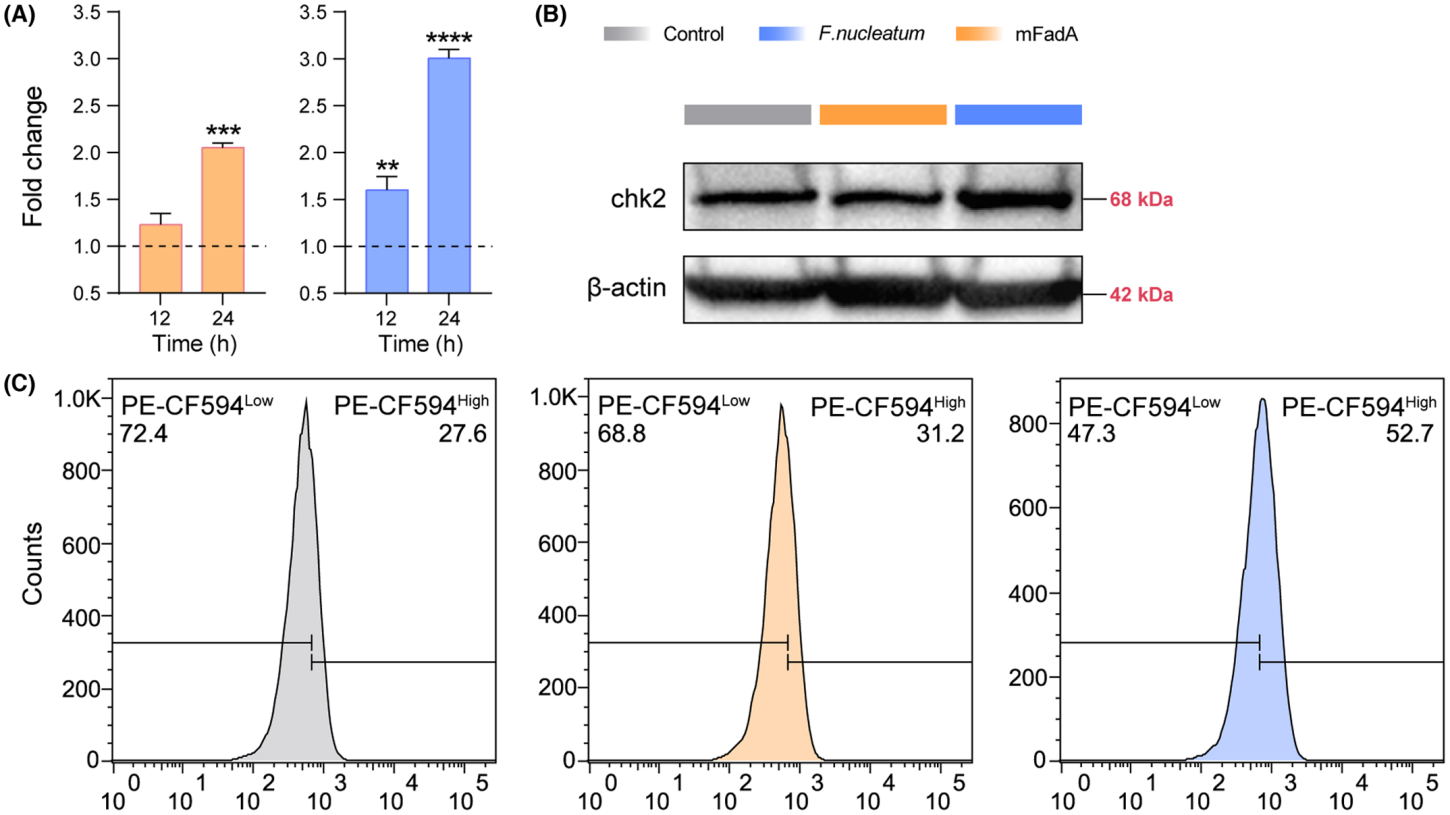
*Fusobacterium nucleatum* regulates the expression of the CHK2 gene in Caco‐2 cells. (A) Quantitative real‐time RT‐PCR assay of CHK2 gene expression in Caco‐2 cells. Based on the comparison with respective control samples, data are expressed as mean with SD. **p < 0.01; ***p < 0.001; ****p < 0.0001. (B) Western blotting results showing the protein expression of Caco‐2 cells. (C) The detection of gene expression changes in Caco‐2 cells by flow cytometry. The cells were preincubated with *F. nucleatum* or mFadA for 24 h prior to the experiments.

As a crucial gene responsible for tumor growth and increased DNA damage in CRC cells, the expression of CHK2 was detected at the levels of transcription and translation.^28^ In the quantitative real‐time RT‐PCR assay, the transcriptional level of CHK2 was increased after a 24‐h preincubation with either *F. nucleatum* or mFadA (Figure 7A). Western blotting exhibited an augmentation of CHK2 expression in the *F. nucleatum* incubation group, but no significant change was observed in the cells treated with mFadA (Figure 7B). Intracellular staining showed a significant variation of CHK2 augmentation in *F. nucleatum* co‐incubated cells to confirm the above results (Figure 7C). Caco‐2 cells in the mFadA group also showed the same trend of CHK2 gene translation as those in the *F. nucleatum* group, although less pronounced.

## 4 DISCUSSION

CRC is often considered a marker of socioeconomic development, since its incidence tends to increase uniformly with ascending Human Development Index (HDI).^2^ In relatively affluent East China, the more Westernized lifestyles and increased lifespan have led to a particularly rapid rise in CRC incidence rate.^6^ Given the increasing importance of the intestinal microbiome in CRC pathogenesis, we performed a comparative analysis of the gut microbiota between local healthy people and corresponding cancer patients and then further investigated the abundance and functional changes in the bacteria of oral origin. Consistent with our results, previous reports have shown a lower community richness in the gut microbiota of CRC patients.^29^ However, we further found that though more bacterial species were detected, the CRC patient group exhibited lower species evenness within the single sample, implying an excessive proliferation of some dominant intestinal bacteria under tumor conditions.

On many levels, the taxonomic and functional compositions of intestinal bacteria were greatly changed in CRC patients, showing a higher abundance of pro‐inflammatory opportunistic bacteria. We identified a series of bacteria contributing to the community disparity between the two groups, which were mainly in phyla *Fusobacteria*, *Proteobacteria*, *Actinobacteria*, and *Firmicutes*. As a relevant biomarker of gut dysbiosis, a higher *Firmicutes*/*Bacteroidetes* ratio is a hallmark of obesity.^30^ Probably because the dysbiosis of intestinal bacteria was an important risk factor for CRC, this ratio was also found to increase remarkably in our patient group.^31^ At the genus level, *Escherichia‐Shigella*, *Fusobacterium*, *Prevotella*, and *Lachnospiracea* could be potential biomarkers for CRC diagnosis. The higher abundance of *Escherichia*, *Shigella*, and *Fusobacterium* in CRC patients has already been confirmed by a number of previous studies.^32^ Meanwhile, *Shigella* was also reported to be over‐represented in the mucosal microbiome of colorectal adenoma patients.^33^ Recognized as the causative agent of human diarrheal disease, *Shigella* might increase host susceptibility to CRC via prolonging the inflammatory response, while the carcinogenic *Escherichia* could produce cytolethal distending toxin and induce double‐strand DNA breaks via its deoxyribonuclease activity.^25^ Comparing healthy subjects with advanced adenoma and CRC patients, the relative abundance of *Fusobacterium* was positively correlated with tumor stages, also remarked as an index to indicate a poor prognosis.^34^ The pro‐inflammatory, immunosuppressive, and tissue‐invasive properties of *Fusobacterium* have been demonstrated by multiple studies.^25^ Through the adhesion of two outer membrane proteins FadA and Fap2, bacteria of this genus could combine with tumor cells and immune cells, affecting various stages of the CRC process.^14^, ^35^

Specific intestinal microbiomes can help regulate the mucosal homeostasis of the host through bacterial metabolism.^25^ As the main producers of endogenous butyrate in the intestinal tract, *Firmicutes* (at the phylum level), *Clostridia* (at the class and order levels), and *Bifidobacteria* (at the genus, family, and order levels) were significantly higher in our 52 healthy volunteers.^29^ Produced by bacterial fermentation, butyrate is an organic acid serving as the major energy source and regulator of proliferation and differentiation for colonic epithelial cells.^36^ Butyrate could not only modulate intestinal barrier function and immune response, but exert an anticancer effect through inhibiting the release of various inflammatory mediators and upregulating P53 gene expression.^29^ Moreover, commensal *Bifidobacterium* has been reported to mitigate intestinal immunopathology in the case of cytotoxic T lymphocyte‐associated antigen‐4 (CTLA‐4) blockade, also promote an antitumor response by the facilitation of anti‐PD‐L1 (programmed cell death 1 ligand 1) efficacy.^25^ At the level of phylum and class, the enriched *Actinobacteria* in healthy controls could be a biomarker. Being a large component of the human intestinal microbiome, some genera under *Actinobacteria* and their secondary metabolites have inhibitory effects on tumor cell lines.^37^ At the genus level, the abundance of *Faecalibacterium* was significantly lower in the CRC group. CRC patients with more abundant *Faecalibacterium* in the gut generally showed a better chemotherapy response and prognosis.^38^ In summary, these bacteria that colonize the intestinal tract of healthy people may play a variety of beneficial roles in preventing inflammation and tumorigenesis. On the contrary, intestinal microbiota as a biomarker has great application prospects for early detection, treatment, and prognosis evaluation of tumors.

The microbial composition in the host gut is susceptible to various environmental factors.^14^ Oral cavity is a major reservoir involved in the formation of intestinal microbiota, but the dissemination of many oral bacteria into the gut often leads to intestinal dysbiosis.^35^ The ectopic intestinal dissemination of multiple oral bacteria, especially pathogenic ones such as *Fusobacterium*, is more profound in colonic adenoma and CRC patients.^39^ These members of the oral microbiota are able to stimulate inflammation by synergistic metabolism, also synthesize volatile sulfur compounds, reactive oxygen species (ROS), and polyamines.^14^ Based on the ectopic abundance in the gut and compositional changes of oral bacteria from previous studies, we further performed taxonomic and functional analyses on six important genera. Concordant with our results, increased *Fusobacterium* population of oral origin was frequently detected in CRC.^32^ However, in the intestines of cancer patients, due to the different trends detected in various relevant studies, changes in the abundance of *Prevotella*, *Ruminococcus*, *Streptococcus*, *Roseburia*, and *Eubacterium* remain elusive.^14^, ^40^ We found a significantly decreased abundance of *Ruminococcus* (55% reduction) and *Prevotella* (82% reduction) in the CRC group. These oral microbes could inhabit the colon in biofilm‐like structures, forming a cooperative polymicrobial network and interacting with tumor tissues.^41^ Previous studies revealed that gender, age, and weight had a potentially strong correlation with the composition of intestinal bacteria, but we found no significant difference in the abundance of six ectopic oral bacteria among several groups.^42^

Through the functional prediction of gut bacteria, our results suggest a slightly evident downregulation of global gene expression in CRC patients. In healthy controls, the functions related to transcription regulation and signal transduction were more abundant, implying defense mechanisms to generate effective protection. In contrast, the CRC group featured richer adhesion, biogenesis, and metabolism functions, especially a number of sucrose, amino sugar, and nucleotide sugar metabolic pathways. As for the six ectopic oral bacteria genera, *Fusobacterium* exhibited the most significant increase in functional expression in CRC patients, while distinctive function and pathway declines were observed in *Prevotella* and *Ruminococcus*. Among the predicted functions, changes in these oral bacteria were mainly involved in energy metabolism and biosynthesis, with *Fusobacterium* showing a particularly significant elevation in glycolytic pathway expression. The increased metabolism of *Fusobacterium* was further confirmed by the prediction of carbohydrate‐related modules. On a diet rich in saturated fatty acid and sugar, intestinal bacteria participate in the production of various carcinogens, such as hydrogen sulfide, ROS, and polyamine.^31^ Produced from trimethylamine, microbial metabolite trimethylamine‐*N*‐oxide (TMAO) promotes the malignant progression of CRC.^43^ Consistent with our results, enhanced bacterial β‐glucuronidase activity has been detected in fecal samples of CRC patients, which could involve the xenobiotic metabolism of the chemotherapeutic drug irinotecan and cause serious side effects.^44^

In view of its significant increase in abundance and important changes in function among CRC patients, we further validated and studied the presence of *Fusobacterium in* the tumor group. Using *Fusobacterium*‐specific primers, bacterial colonization was detected in the colonic biopsy specimens of the CRC patients. Among several of the most common *Fusobacterium* species clinically causing intestinal infection, *F. nucleatum* subsp. *animalis* exhibited the closest genetic relationship to our tissue samples.^45^ Similarly, a previous study in Shanghai, China, also found that *animalis* was consistently the most prevalent subspecies in the intestines of local CRC patients, showing differences from many other regions of the world.^46^

The invasiveness of *F. nucleatum* varies greatly among different strains.^47^ To date, few studies targeting the pathogenicity of *F. nucleatum* in CRC have involved the *animalis* subspecies.^28^, ^48^, ^49^ Subsp. *animalis* could interact directly with monocytes, recruiting other immunoregulatory cells by CCL20 signaling and then promoting CRC progression.^50^ Mediated by LPS, interaction of the subspecies with Siglec‐7, which is expressed by various immune cells, was also demonstrated.^51^ Here, we further verified the interaction of *F. nucleatum* subsp. *animalis* ATCC 51191 with human intestinal epithelium and tumor cells. Due to the presence of vacuoles and blurred edges in Caco‐2 cells during adherent growth, the HCT 116 cell line was used instead for the bacterial adherence detection in SPRi experiments. A driver‐passenger model was established for the role of the gut microbiota in CRC, in which directly carcinogenic bacteria (drivers) and opportunistic bacteria in the tumor‐associated microenvironment (passengers) play different roles in multiple stages of the cancer.^52^ With its significantly higher selectivity and proliferation‐promoting effect on tumor cells, our results reaffirmed the role of *F. nucleatum* as an important passenger bacterium. Interestingly, the role of this bacterium in CRC could be intricate. *F. nucleatum* strains isolated from inflamed tissues of inflammatory bowel disease (IBD) patients were found to be more invasive than those from normal tissues, suggesting its potential as a commensal‐turned pathogen with variable virulence.^47^ Since IBD has also been identified as an important risk factor for CRC, the possible mechanism of *F. nucleatum* in causing CRC deserves further elucidation.^53^

FadA, a vital adhesin on the surface of *F. nucleatum*, was identified to confer acid tolerance and function as a scaffold for biofilm formation, also the most characteristic virulence factor of the bacterium.^49^ FadA exists in two forms, the intact pre‐FadA (129 aa) and the secreted mFadA (111 aa), constituting an active complex FadAc altered. Through its function of binding and invading host cells, FadA was reported to play a primary role in CRC tumor growth stimulated by *F. nucleatum*.^49^ In our study, secreted mFadA was used to compare with the effect of *F. nucleatum* subsp. *animalis* on CRC cells. On the whole, mFadA could enhance the viability and proliferation of tumor cells, but the effect was significantly lower than that of the bacterium. *F. nucleatum* was found to produce amyloid‐like FadA only under stress and disease conditions, not in healthy tissues.^49^ Moreover, compared with healthy controls, the presence of FadA gene was significantly increased in ulcerative colitis (UC) and CRC patients.^54^ It is worth mentioning, that though FadA is more likely to adhere to inflammatory and tumor tissues, the FadA‐mediated binding also exist on normal cells. Increased colonization of *F. nucleatum* in normal tissues may predisposes the host to adenoma, and the carcinogenic effect would be accelerated when mutations occur.

Checkpoint kinase 2 (CHK2), an important multifunctional enzyme associated with cell cycle arrest and apoptosis caused by DNA damage, has been implicated in multiple tumors such as prostate cancer and liver cancer.^55^ During CRC progression, CHK2 plays a significant role in tumor growth and increased DNA damage in cancer cells, and *F. nucleatum* was found to promote its gene upregulation.^28^ Thus, we detected CHK2 expressions in Caco‐2 cells stimulated by subsp. *animalis* and mFadA protein for verification, suggesting a potential target for CRC treatment. Probably because of the lack of the complete FadAc composite structure, cells in the mFadA group showed an upregulated trend of CHK2 expression, though less pronounced than that in the bacteria‐treated cells.^28^

With the continuous changes in lifestyles, a parallel evolutionary history for humans in different regions and their gut bacterial lineages has developed.^56^ The present study confirmed previous hypotheses that CRC is associated with many microbial composition alterations in the human gut, also extended the knowledge of characteristic bacterial changes and functional differences in the East China population. However, limitations exist in this work. For bacterial functional analysis, the clinical sample size used in shotgun metagenomic sequencing could be increased. In addition to the role of *Fusobacterium* that we validated, it is still unknown whether the alterations in other ectopic oral bacteria are a consequence of the CRC process or somehow involved in its pathogenesis. In future research, quantitative metagenomics analysis could be expanded to include more gastrointestinal disorders such as IBD and colon polyps for cross‐disease comparisons. The collective contribution of multiple bacteria or bacteria with other intestinal microorganisms, such as viruses and parasites, in CRC patients also needs to be clarified. Finally, in‐depth experiments should refine the biological function of the oral‐origin bacteria in the gut microbiome, especially *F. nucleatum* subsp. *animalis*, throughout the cancer process. Future studies could verify the presence and distribution of amyloid‐like FadA by performing immunoassays on colonic biopsy specimens from relevant patients with ethical approval, and use it as a possible intervention goal for *F. nucleatum*‐mediated diseases. We hope that our cohort study could offer some guidance on the use of specific gut bacteria as biomarkers for CRC risk prediction, and also as targets for prevention and treatment of cancer, or even various intestinal diseases.

## AUTHOR CONTRIBUTIONS

**Hongze Zhang:** Data curation (equal); formal analysis (equal); visualization (equal); writing – original draft (equal). **Kai Jin:** Data curation (equal); investigation (equal); resources (equal). **Kunlong Xiong:** Investigation (equal). **Wenwen Jing:** Investigation (equal). **Zhen Pang:** Investigation (equal). **Meng Feng:** Data curation (equal); investigation (equal); writing – review and editing (equal). **Xunjia Cheng:** Conceptualization (equal); formal analysis (equal); methodology (equal); writing – review and editing (equal).

## FUNDING INFORMATION

This work was supported by the National Natural Science Foundation of China (81630057) and the National Key Research and Development Program of China (2018YFA0507304).

## CONFLICT OF INTEREST STATEMENT

All the authors declared no conflicts of interest with respect to the research, authorship, and publication of this article.

## ETHICS AND CONSENT STATEMENT

All procedures followed were in accordance with the ethical standards of the Medical Ethics Committee of Huadong Hospital affiliated to Fudan University (permit AF16‐20170052) and with the *Helsinki Declaration* of 1975, as revised in 2000 (5). Informed consent was obtained from all patients for being included in the study.

## Supporting information


Figures S1–S5.

Supplementary Methods

Tables S1–S6.

## Data Availability

Detailed data can be obtained by contacting the corresponding author.

## References

[1] Arnold M , Abnet CC , Neale RE , et al. Global burden of 5 major types of gastrointestinal cancer. Gastroenterology. 2020;159(1):335‐349.e15. 32247694 10.1053/j.gastro.2020.02.068 PMC8630546

[2] Sung H , Ferlay J , Siegel RL , et al. Global cancer statistics 2020: GLOBOCAN estimates of incidence and mortality worldwide for 36 cancers in 185 countries. CA Cancer J Clin. 2021;71(3):209‐249. 33538338 10.3322/caac.21660

[3] Arnold M , Sierra MS , Laversanne M , Soerjomataram I , Jemal A , Bray F . Global patterns and trends in colorectal cancer incidence and mortality. Gut. 2017;66(4):683‐691. 26818619 10.1136/gutjnl-2015-310912

[4] Sun D , Cao M , Li H , He S , Chen W . Cancer burden and trends in China: a review and comparison with Japan and South Korea. Chin J Cancer Res. 2020;32(2):129‐139. 32410791 10.21147/j.issn.1000-9604.2020.02.01 PMC7219092

[5] Cao M , Li H , Sun D , Chen W . Cancer burden of major cancers in China: a need for sustainable actions. Cancer Commun (Lond). 2020;40(5):205‐210. 32359212 10.1002/cac2.12025 PMC7667573

[6] Cao W , Chen HD , Yu YW , Li N , Chen WQ . Changing profiles of cancer burden worldwide and in China: a secondary analysis of the global cancer statistics 2020. Chin Med J (Engl). 2021;134(7):783‐791. 33734139 10.1097/CM9.0000000000001474 PMC8104205

[7] Lim ES , Wang D , Holtz LR . The bacterial microbiome and virome milestones of infant development. Trends Microbiol. 2016;24(10):801‐810. 27353648 10.1016/j.tim.2016.06.001

[8] Jackson DN , Theiss AL . Gut bacteria signaling to mitochondria in intestinal inflammation and cancer. Gut Microbes. 2020;11(3):285‐304. 30913966 10.1080/19490976.2019.1592421 PMC7524274

[9] Zhou C , Zhao H , Xiao XY , et al. Metagenomic profiling of the pro‐inflammatory gut microbiota in ankylosing spondylitis. J Autoimmun. 2020;107:102360. 31806420 10.1016/j.jaut.2019.102360

[10] Zhu F , Ju Y , Wang W , et al. Metagenome‐wide association of gut microbiome features for schizophrenia. Nat Commun. 2020;11(1):1612. 32235826 10.1038/s41467-020-15457-9 PMC7109134

[11] Park CH , Eun CS , Han DS . Intestinal microbiota, chronic inflammation, and colorectal cancer. Intest Res. 2018;16(3):338‐345. 30090032 10.5217/ir.2018.16.3.338 PMC6077304

[12] Carpenter GH . The secretion, components, and properties of saliva. Annu Rev Food Sci Technol. 2013;4:267‐276. 23464573 10.1146/annurev-food-030212-182700

[13] Pedersen AML , Sorensen CE , Proctor GB , Carpenter GH , Ekstrom J . Salivary secretion in health and disease. J Oral Rehabil. 2018;45(9):730‐746. 29878444 10.1111/joor.12664

[14] Koliarakis I , Messaritakis I , Nikolouzakis TK , Hamilos G , Souglakos J , Tsiaoussis J . Oral bacteria and intestinal dysbiosis in colorectal cancer. Int J Mol Sci. 2019;20(17):4146. 31450675 10.3390/ijms20174146 PMC6747549

[15] Abed J , Maalouf N , Manson AL , et al. Colon cancer‐associated *Fusobacterium nucleatum* may originate from the oral cavity and reach colon tumors via the circulatory system. Front Cell Infect Microbiol. 2020;10:400. 32850497 10.3389/fcimb.2020.00400 PMC7426652

[16] Hajishengallis G . Periodontitis: from microbial immune subversion to systemic inflammation. Nat Rev Immunol. 2015;15(1):30‐44. 25534621 10.1038/nri3785 PMC4276050

[17] Ahn J , Chen CY , Hayes RB . Oral microbiome and oral and gastrointestinal cancer risk. Cancer Causes Control. 2012;23(3):399‐404. 22271008 10.1007/s10552-011-9892-7 PMC3767140

[18] Tilg H , Adolph TE , Gerner RR , Moschen AR . The intestinal microbiota in colorectal cancer. Cancer Cell. 2018;33(6):954‐964. 29657127 10.1016/j.ccell.2018.03.004

[19] Dong J , Li Y , Xiao H , et al. Oral microbiota affects the efficacy and prognosis of radiotherapy for colorectal cancer in mouse models. Cell Rep. 2021;37(4):109886. 34706245 10.1016/j.celrep.2021.109886

[20] Bharti R , Grimm DG . Current challenges and best‐practice protocols for microbiome analysis. Brief Bioinform. 2021;22(1):178‐193. 31848574 10.1093/bib/bbz155 PMC7820839

[21] Escobar‐Zepeda A , Vera‐Ponce de Leon A , Sanchez‐Flores A . The road to metagenomics: from microbiology to DNA sequencing technologies and bioinformatics. Front Genet. 2015;6:348. 26734060 10.3389/fgene.2015.00348 PMC4681832

[22] Ma Y , Zhang Y , Xiang J , et al. Metagenome analysis of intestinal bacteria in healthy people, patients with inflammatory bowel disease and colorectal cancer. Front Cell Infect Microbiol. 2021;11:599734. 33738265 10.3389/fcimb.2021.599734 PMC7962608

[23] Feng M , Zhang Y , Zhou H , et al. Single‐cell RNA sequencing reveals that the switching of the transcriptional profiles of cysteine‐related genes alters the virulence of *Entamoeba histolytica* . mSystems. 2020;5(6):e01095‐20. 33361325 10.1128/mSystems.01095-20 PMC7762796

[24] Jing W , Wang Y , Yang Y , et al. Time‐resolved digital immunoassay for rapid and sensitive quantitation of procalcitonin with plasmonic imaging. ACS Nano. 2019;13(8):8609‐8617. 31276361 10.1021/acsnano.9b02771 PMC7008466

[25] Wong SH , Yu J . Gut microbiota in colorectal cancer: mechanisms of action and clinical applications. Nat Rev Gastroenterol Hepatol. 2019;16(11):690‐704. 31554963 10.1038/s41575-019-0209-8

[26] Uchino Y , Goto Y , Konishi Y , et al. Colorectal cancer patients have four specific bacterial species in oral and gut microbiota in common—a metagenomic comparison with healthy subjects. Cancers (Basel). 2021;13(13):3332. 34283063 10.3390/cancers13133332 PMC8268706

[27] El‐Said WA , Yea CH , Kim H , Oh BK , Choi JW . Cell‐based chip for the detection of anticancer effect on HeLa cells using cyclic voltammetry. Biosens Bioelectron. 2009;24(5):1259‐1265. 18782663 10.1016/j.bios.2008.07.037

[28] Guo P , Tian Z , Kong X , et al. FadA promotes DNA damage and progression of *Fusobacterium nucleatum*‐induced colorectal cancer through up‐regulation of chk2. J Exp Clin Cancer Res. 2020;39(1):202. 32993749 10.1186/s13046-020-01677-w PMC7523382

[29] Zhang YK , Zhang Q , Wang YL , et al. A comparison study of age and colorectal cancer‐related gut bacteria. Front Cell Infect Microbiol. 2021;11:606490. 33996615 10.3389/fcimb.2021.606490 PMC8121496

[30] Magne F , Gotteland M , Gauthier L , et al. The firmicutes/bacteroidetes ratio: a relevant marker of gut dysbiosis in obese patients? Nutrients. 2020;12(5):1474. 32438689 10.3390/nu12051474 PMC7285218

[31] Janney A , Powrie F , Mann EH . Host‐microbiota maladaptation in colorectal cancer. Nature. 2020;585(7826):509‐517. 32968260 10.1038/s41586-020-2729-3

[32] Luo K , Zhang Y , Xv C , et al. *Fusobacterium nucleatum*, the communication with colorectal cancer. Biomed Pharmacother. 2019;116:108988. 31112873 10.1016/j.biopha.2019.108988

[33] Shen XJ , Rawls JF , Randall T , et al. Molecular characterization of mucosal adherent bacteria and associations with colorectal adenomas. Gut Microbes. 2010;1(3):138‐147. 20740058 10.4161/gmic.1.3.12360 PMC2927011

[34] Clos‐Garcia M , Garcia K , Alonso C , et al. Integrative analysis of fecal metagenomics and metabolomics in colorectal cancer. Cancers (Basel). 2020;12(5):1142. 32370168 10.3390/cancers12051142 PMC7281174

[35] Flemer B , Warren RD , Barrett MP , et al. The oral microbiota in colorectal cancer is distinctive and predictive. Gut. 2018;67(8):1454‐1463. 28988196 10.1136/gutjnl-2017-314814 PMC6204958

[36] Bridgeman SC , Northrop W , Melton PE , Ellison GC , Newsholme P , Mamotte CDS . Butyrate generated by gut microbiota and its therapeutic role in metabolic syndrome. Pharmacol Res. 2020;160:105174. 32860943 10.1016/j.phrs.2020.105174

[37] Rangan KJ , Hang HC . Biochemical mechanisms of pathogen restriction by intestinal bacteria. Trends Biochem Sci. 2017;42(11):887‐898. 28927699 10.1016/j.tibs.2017.08.005 PMC6038137

[38] Yang Y , Misra BB , Liang L , et al. Integrated microbiome and metabolome analysis reveals a novel interplay between commensal bacteria and metabolites in colorectal cancer. Theranostics. 2019;9(14):4101‐4114. 31281534 10.7150/thno.35186 PMC6592169

[39] Schmidt TS , Hayward MR , Coelho LP , et al. Extensive transmission of microbes along the gastrointestinal tract. Elife. 2019;8:e42693. 30747106 10.7554/eLife.42693 PMC6424576

[40] Long X , Wong CC , Tong L , et al. *Peptostreptococcus anaerobius* promotes colorectal carcinogenesis and modulates tumour immunity. Nat Microbiol. 2019;4(12):2319‐2330. 31501538 10.1038/s41564-019-0541-3

[41] Alvarez S , Leiva‐Sabadini C , Schuh C , Aguayo S . Bacterial adhesion to collagens: implications for biofilm formation and disease progression in the oral cavity. Crit Rev Microbiol. 2022;48(1):83‐95. 34270375 10.1080/1040841X.2021.1944054

[42] Byrd AL , Liu M , Fujimura KE , et al. Gut microbiome stability and dynamics in healthy donors and patients with non‐gastrointestinal cancers. J Exp Med. 2021;218(1):e20200606. 33175106 10.1084/jem.20200606 PMC7664509

[43] Zou S , Fang L , Lee MH . Dysbiosis of gut microbiota in promoting the development of colorectal cancer. Gastroenterol Rep (Oxf). 2018;6(1):1‐12. 29479437 10.1093/gastro/gox031 PMC5806407

[44] Wallace BD , Wang H , Lane KT , et al. Alleviating cancer drug toxicity by inhibiting a bacterial enzyme. Science. 2010;330(6005):831‐835. 21051639 10.1126/science.1191175 PMC3110694

[45] Yeoh YK , Chen Z , Wong MCS , et al. Southern Chinese populations harbour non‐nucleatum Fusobacteria possessing homologues of the colorectal cancer‐associated FadA virulence factor. Gut. 2020;69(11):1998‐2007. 32051205 10.1136/gutjnl-2019-319635 PMC7569397

[46] Bi D , Zhu Y , Gao Y , et al. A newly developed PCR‐based method revealed distinct *Fusobacterium nucleatum* subspecies infection patterns in colorectal cancer. J Microbial Biotechnol. 2021;14(5):2176‐2186. 10.1111/1751-7915.13900 PMC8449656 34309194

[47] Han YW . *Fusobacterium nucleatum*: a commensal‐turned pathogen. Curr Opin Microbiol. 2015;23:141‐147. 25576662 10.1016/j.mib.2014.11.013 PMC4323942

[48] Li X , Huang J , Yu T , et al. *Fusobacterium nucleatum* promotes the progression of colorectal cancer through Cdk5‐activated Wnt/beta‐catenin signaling. Front Microbiol. 2020;11:545251. 33488528 10.3389/fmicb.2020.545251 PMC7815597

[49] Meng Q , Gao Q , Mehrazarin S , et al. *Fusobacterium nucleatum* secretes amyloid‐like FadA to enhance pathogenicity. EMBO Rep. 2021;22(7):e52891. 34184813 10.15252/embr.202152891 PMC8406402

[50] Wang N , Fang JY . *Fusobacterium nucleatum*, a key pathogenic factor and microbial biomarker for colorectal cancer. Trends Microbiol. 2022;31(2):159‐172. 36058786 10.1016/j.tim.2022.08.010

[51] Lamprinaki D , Garcia‐Vello P , Marchetti R , et al. Siglec‐7 mediates immunomodulation by colorectal cancer‐associated *Fusobacterium nucleatum* ssp. animalis. Front Immunol. 2021;12:744184. 34659241 10.3389/fimmu.2021.744184 PMC8517482

[52] Tjalsma H , Boleij A , Marchesi JR , Dutilh BE . A bacterial driver‐passenger model for colorectal cancer: beyond the usual suspects. Nat Rev Microbiol. 2012;10(8):575‐582. 22728587 10.1038/nrmicro2819

[53] Shah SC , Itzkowitz SH . Colorectal cancer in inflammatory bowel disease: mechanisms and management. Gastroenterology. 2022;162(3):715‐730.e3. 34757143 10.1053/j.gastro.2021.10.035 PMC9003896

[54] Li DH , Li ZP , Yan Z , et al. Fecal *Fusobacterium nucleatum* harbored virulence gene fadA are associated with ulcerative colitis and clinical outcomes. Microb Pathog. 2021;157:104964. 34022363 10.1016/j.micpath.2021.104964

[55] Lulli M , Del Coco L , Mello T , et al. DNA damage response protein CHK2 regulates metabolism in liver cancer. Cancer Res. 2021;81(11):2861‐2873. 33762357 10.1158/0008-5472.CAN-20-3134

[56] Suzuki TA , Fitzstevens JL , Schmidt VT , et al. Codiversification of gut microbiota with humans. Science. 2022;377(6612):1328‐1332. 36108023 10.1126/science.abm7759 PMC10777373

