# Supplementary material for: Disease‐associated gut microbiome and critical metabolomic alterations in patients with colorectal cancer
Source: Cancer Med. 2023 Jun 1;12(14):15720–35. doi: 10.1002/cam4.6194 (PMC10417192; doi:10.1002/cam4.6194)
Supplement: Supplementary file 1 — Figures S1–S5. [file CAM4-12-15720-s002.docx]

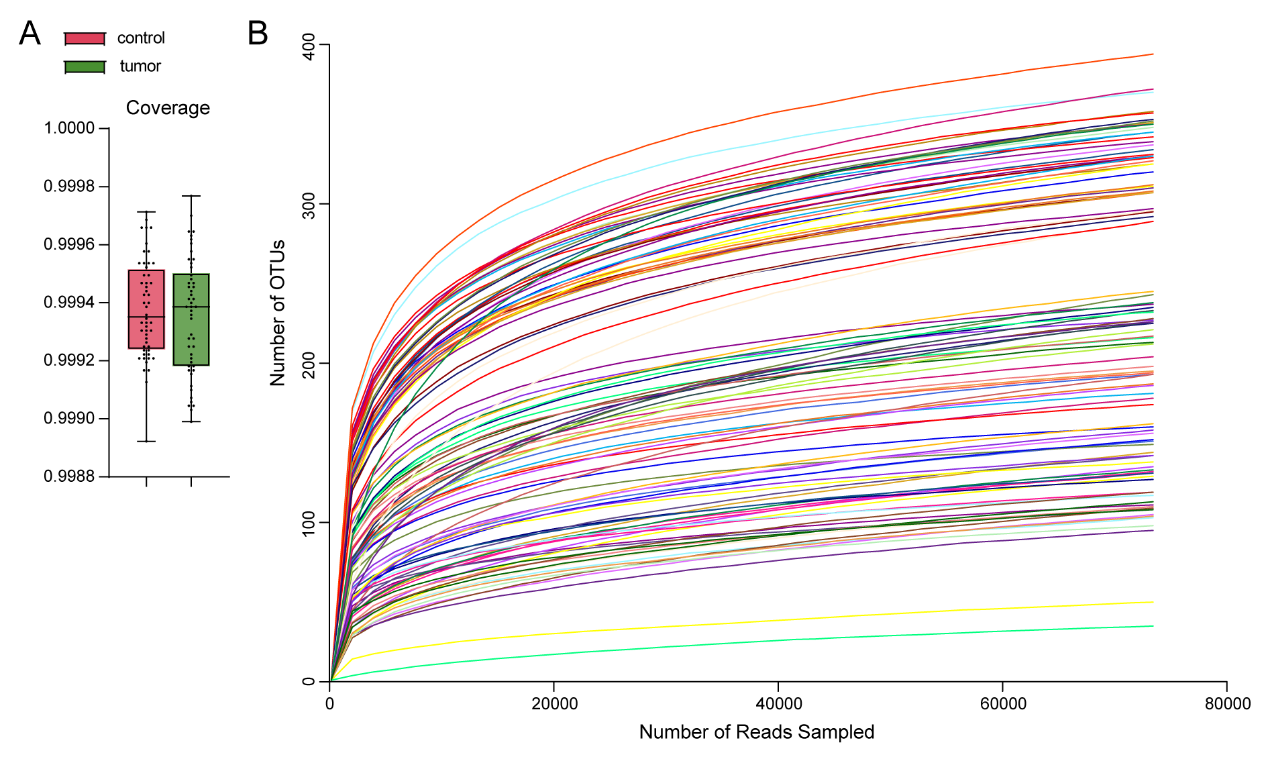
 **Fig. S1** Reflection of the real bacterial situation in the sample. (A) The coverage ratio of the library. (B) Rarefaction curves for each sample.


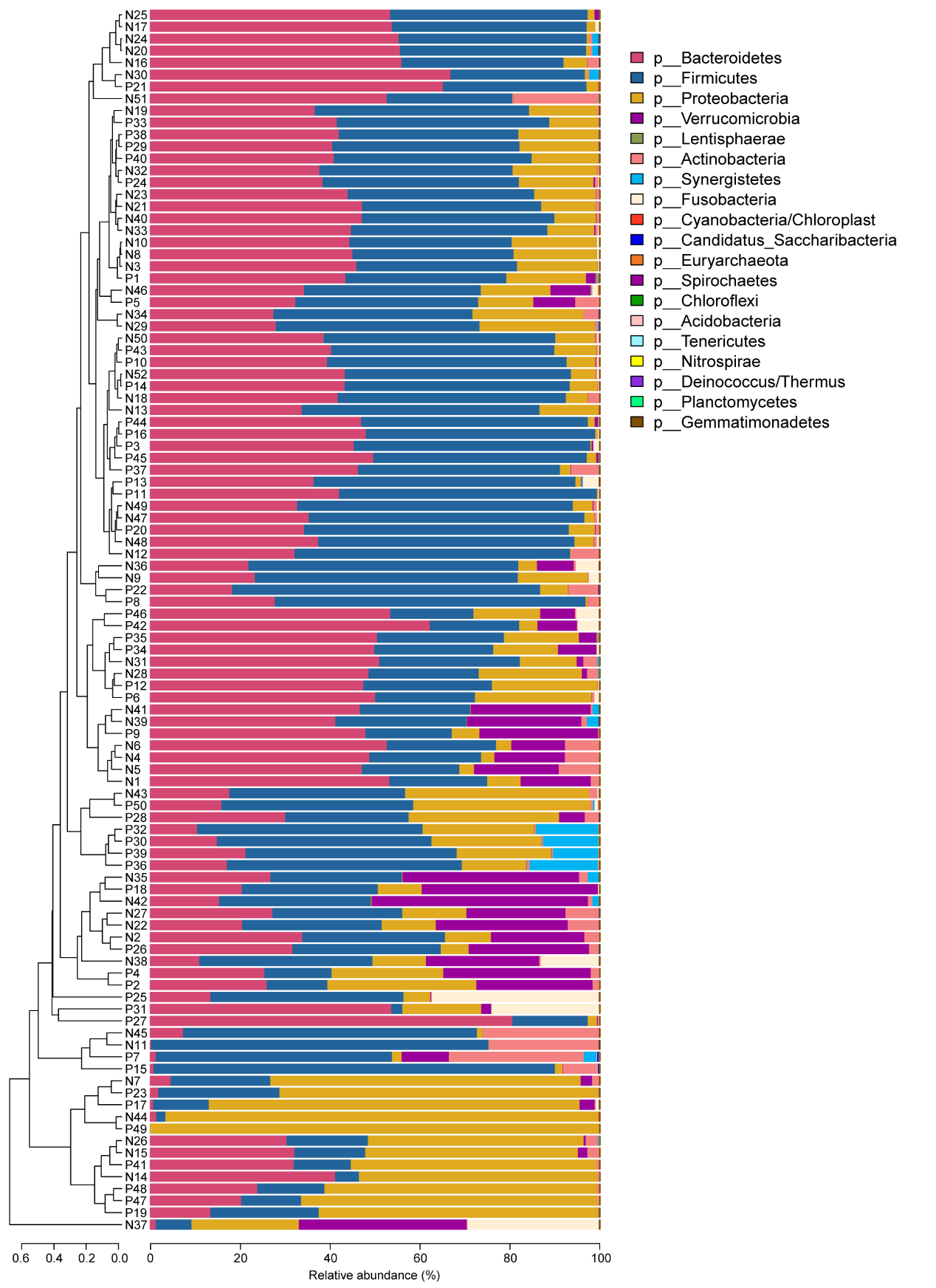
**Fig. S2** Bacterial composition at the phylum level for each sample. The figure used 16S rDNA sequencing data from 102 samples.


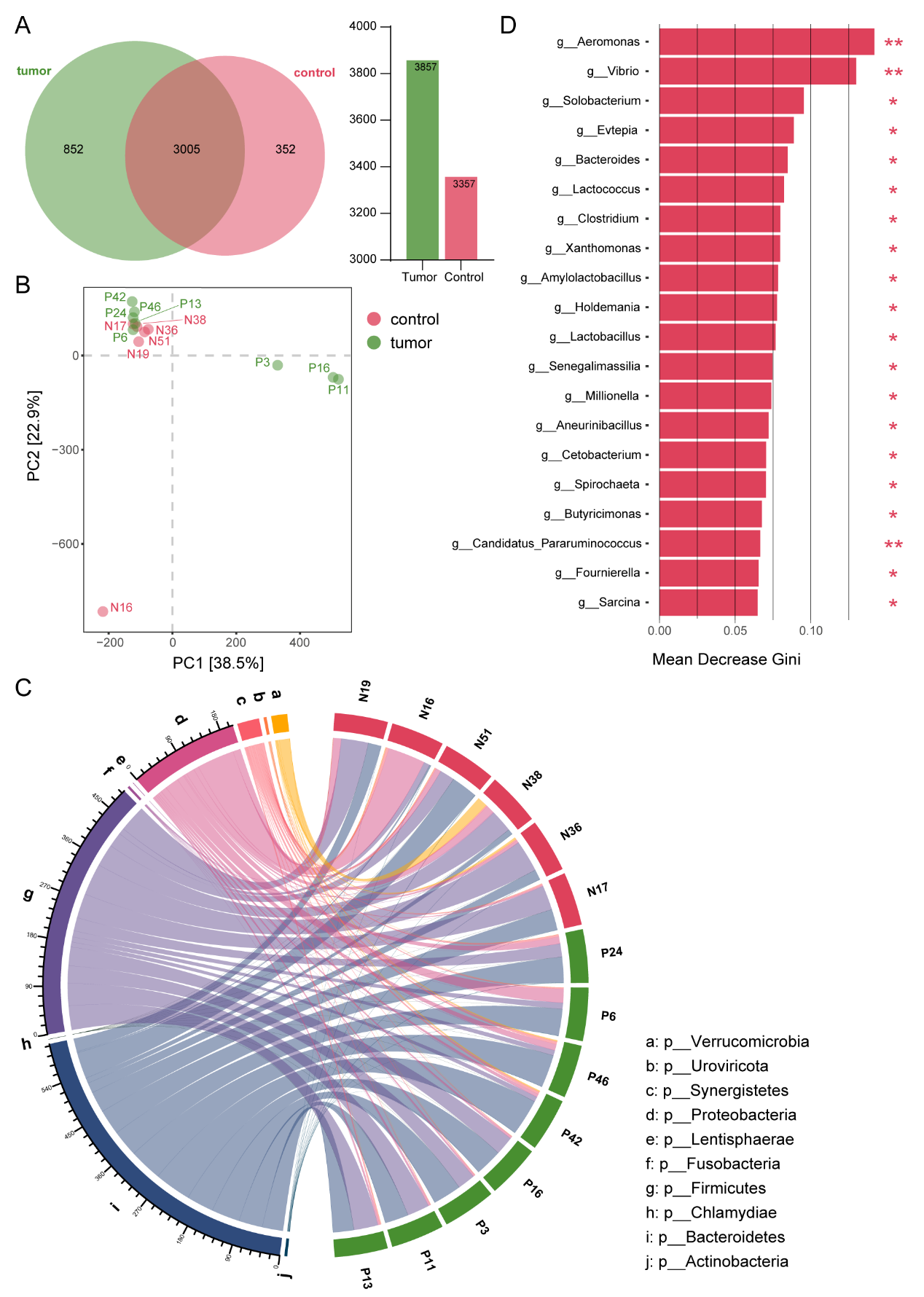
**Fig. S3** Taxonomic characteristics of the two similar sample groups. (A) The common and unique OTUs between eight CRC patients and six healthy volunteers. (B) Principal component analysis showed differences in bacterial composition between the two sample groups. (C) A Circos diagram showing the proportion of dominant phyla in each sample. (D) The top 20 genus sources of differences between groups showed by Mean Decrease Gini coefficient from Random Forest analysis. * P<0.05, ** P<0.01. Shotgun metagenomic sequencing data was used in the figure.


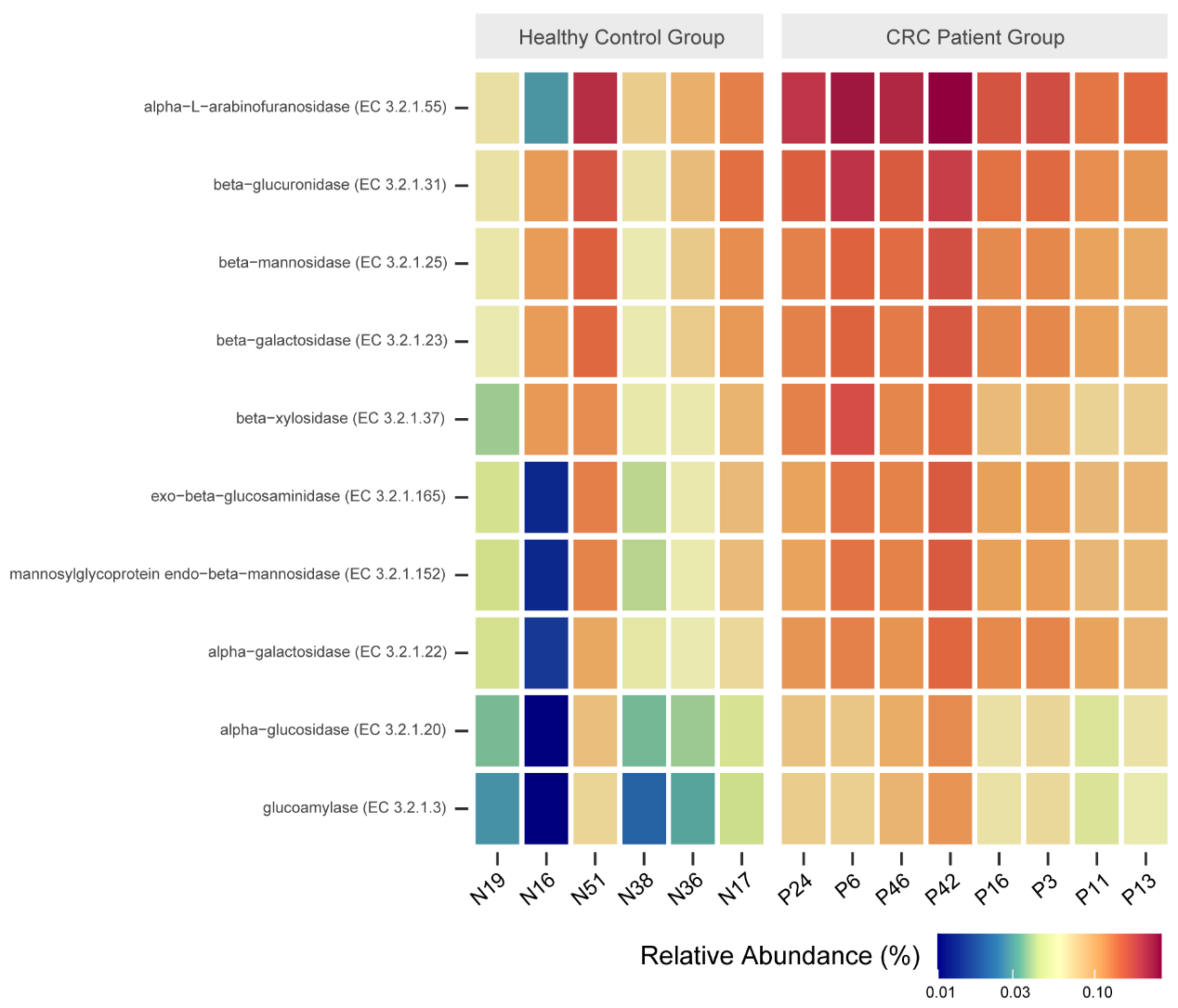
**Fig. S4** Relative abundances of the top ten carbohydrate-related enzymes among selected samples. Shotgun metagenomic sequencing data was used in the figure.


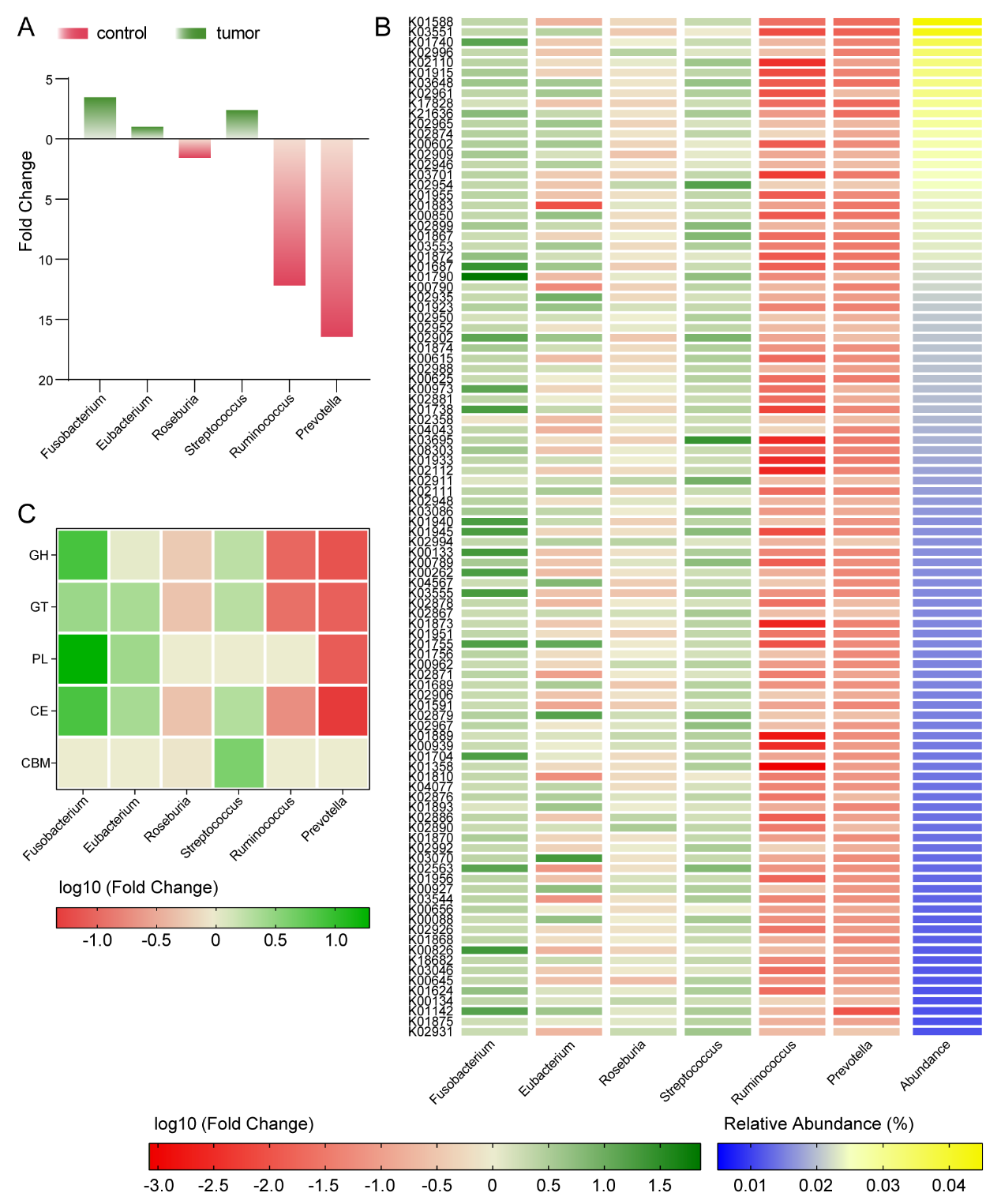
**Fig. S5** Functional differences of the six oral bacteria between the two sample groups. (A) Fold changes in the global gene expression of six oral bacterial genera. (B) After comparison with the KEGG database, differences in the expression of the 100 most abundant orthologous genes among the oral bacteria are shown by a heat map. (C) After comparison with the CAZy database, differences in carbohydrate metabolism among the oral bacteria are shown by a heat map. The red color represents a higher expression in the healthy group, while green represents a higher expression in the CRC group. Shotgun metagenomic sequencing data was used in the figure.
