# Supplementary material for: Disease‐associated gut microbiome and critical metabolomic alterations in patients with colorectal cancer
Source: Cancer Med. 2023 Jun 1;12(14):15720–35. doi: 10.1002/cam4.6194 (PMC10417192; doi:10.1002/cam4.6194)
Supplement: Supplementary file 2 — Supplementary Methods [file CAM4-12-15720-s001.docx]

**Supporting Methods**

**Detection of bacterial DNA from FFPE sections**

To detect the colonization of *Fusobacterium* in tumor tissues, formalin-fixed and paraffin-embedded (FFPE) colonic biopsy specimens of the eight CRC patients were obtained from the archive of Huadong hospital with IRB approval. Following the manufacturer's instructions, DNA extraction was performed with a QIAamp DNA FFPE Tissue kit (Qiagen, Hilden, Germany). PCR trials were conducted using optimized *Fusobacterium*-specific 16S rDNA forward (5'-ACCAGCGTTTGACATCTTAGGA-3') and reverse (5'-CTAATCACACCCTCGGAGCATC-3') primers. The amplification cycling conditions were as follows: 30 s at 95 °C for denaturation, 30 s at 50 °C for annealing, 30 s at 72 °C for extension, and 10 min at 72 °C for the final extension after 40 cycles. Due to the limited number of initial templates, all the products of the first round PCR were cleaned up, followed by dissolution and addition into a second round PCR system as templates.

Final products were cloned into pET-19b vectors (Novagen, Darmstadt, Germany), and the cloned plasmids were transformed into *E. coli* JM109 competent cells (Weidibio, Shanghai, China). The amplified plasmids were then extracted and sequenced.

**Bacterial strains and cell cultures**

*Fusobacterium nucleatum* subsp. *animalis* was purchased from the ATCC (#51191). Under anaerobic conditions, *F. nucleatum* 51191 was grown at 37 °C in Columbia broth with the supplement of 5 μg/ml hemin and 1 μg/ml menadione. During the experiment, 16S rDNA sequencing of the bacteria was performed every month to ensure a lack of contamination.

Human intestinal epithelial cell lines Caco-2 and CCD 841 CoN (purchased from ATCC) were cultured with Eagle's Minimum Essential Medium (MEM; Corning, Manassas, VA, USA) supplemented with 10% fetal bovine serum (HyClone Laboratories, Logan, UT, USA), while HCT 116 was cultured in Dulbecco’s Modified Eagle’s Medium (DMEM; Corning, Manassas, VA, USA). After the addition of 2 mM L-glutamine (Gibco, Grand Island, NY, USA) and 1 mM sodium pyruvate (Gibco, Grand Island, NY, USA), cells were grown in a 5% CO_2_ incubator maintained at 37 °C.

**Expression and purification of recombinant mFadA protein**

Full-length FadA (GenBank accession no. AAY47043.1) without the signal peptide (first 18 aa) was cloned into pET-19b vectors (Novagen, Darmstadt, Germany). *E. coli* BL21 Star(DE3)pLysS competent cells (Weidibio, Shanghai, China) were transformed with the cloned plasmids, then cultured in LB medium containing 100 μg/ml ampicillin.

For the expression of recombinant mFadA, bacteria were induced with isopropyl-β-d-thiogalactopyranoside (Amresco, Solon, OH, USA) at a final concentration of 1 mM. After incubation at 37 °C for 2—3 h, bacteria were harvested and sonicated in binding buffer (20 mM Tris-HCl [pH 7.9], 0.5 M NaCl, 5 mM imidazole) with NP-40 and phenylmethanesulfonyl fluoride (PMSF, final concentration of 1 mM). The supernatants were further purified using a Ni-NTA (nitrilotriacetic acid) His-Bind resin kit (Novagen, Darmstadt, Germany) and filtrated via a 0.22-µm membrane.

A Toxin Sensor Gel Clot Endotoxin Assay Kit (Genscript, Nanjing, China) was applied to detect the endotoxin levels of recombinant proteins, which conform to the national standard of the People’s Republic of China for medical products (GB/t14233.2-2005).

**Cell Cycle Analysis**

After propidium iodide (PI) staining, flow cytometry was performed for cell cycle analysis using a BD FACSCalibur Flow Cytometer. Caco-2 cells were planted into 6-well plates (4 × 10^5^ cells per well) and co-incubated with *F. nucleatum* (MOI=20) or mFadA (5 μg/ml) at 37 °C for 24 h. After harvesting and washing the cells, 70% ethanol was used for fixation and permeabilization. RNaseA (1 g/liter) and PI (0.25 g/liter) were successively added for DNA staining. The number of cells at different stages of the cell cycle was counted for proportion calculations.

**Electrochemical behavior determined by cyclic voltammetry**

Cyclic voltammetry experiments were performed on a BAS100A system (Bioanalytical Systems). The three-electrode system comprised a cell-based chip (working electrode), a platinum wire (auxiliary electrode), and Ag/AgCl (reference electrode). To study the changes in electrical properties affected by *F. nucleatum*, Caco-2 cells were lysed in situ with 1% NP-40 after co-incubation with *F. nucleatum* (MOI=20) or mFadA protein (5 μg/ml). PBS was used as the electrolyte at a scan rate of 0.1 V/s.

**Western blot analysis**

Western blotting was implemented to confirm CHK2 gene expression in Caco-2 cells. After co-incubation with *F. nucleatum* (MOI=50) or mFadA (10 μg/ml) for 24 h, protein samples of the cells were pelleted by centrifugation and separated on 10% polyacrylamide gels, then electrotransferred onto polyvinylidene difluoride membranes (General Electric Co., Schenectady, NY, USA). Membranes were blocked with 5% bovine serum albumin in PBS and incubated with the following primary antibodies: anti-CHK2 monoclonal antibody (Abcam, Ab109413; Cambridge, United Kingdom) and anti-β-actin antibody (Cell Signaling Technology, #3700; Boston, MA, USA). The secondary antibodies were horseradish peroxidase (HRP)-conjugated goat anti-mouse IgG H&L (Abcam, Ab6789) and HRP-conjugated goat anti-rabbit IgG H&L (Abcam, Ab6721). An enhanced chemiluminescence (ECL) Western Blotting Substrate Kit (Tanon, Shanghai, China) was used for the detection of proteins.
