# Supplementary material for: Disease‐associated gut microbiome and critical metabolomic alterations in patients with colorectal cancer
Source: Cancer Med. 2023 Jun 1;12(14):15720–35. doi: 10.1002/cam4.6194 (PMC10417192; doi:10.1002/cam4.6194)
Supplement: Supplementary file 3 — Tables S1–S6. [file CAM4-12-15720-s003.docx]

**Table S1** Primers used in quantitative real-time RT-PCR.

| **Amplified gene** | | **Primer sequence (5' - 3')** |
| --- | --- | --- |
| β-catenin | S | AGCTTCCAGACACGCTATCAT |
|  | AS | CGGTACAACGAGCTGTTTCTAC |
| chk2 | S | TCTCGGGAGTCGGATGTTGAG |
|  | AS | CCTGAGTGGACACTGTCTCTAA |
| Cyclin D1 | S | ACAAACTGTTTTGAAAATCCA |
|  | AS | CGAGTCATTGCATACTGTCC |
| GAPDH | S | TCACCACCATGGAGAAGGC |
|  | AS | GCTAAGCAGTTGGTGGTGCA |

All primers were synthesized by Invitrogen company.

**Table S2** Demographic characteristics of the two cohorts.

| **Characteristics** | **Normal control** | **CRC patients** | **Summation** |
| --- | --- | --- | --- |
| **Gender** | | | |
| Males | 28 (54%) | 33 (66%) | 61 (60%) |
| Females | 24 (46%) | 17 (34%) | 41 (40%) |
| **Age (Year)** | | | |
| Mean ± SD | 54.13±10.21 | 65.32±10.49 | 59.62±10.39 |
| ≤ 65 | 40 (77%) | 23 (46%) | 63 (62%) |
| ＞65 | 12 (23%) | 27 (54%) | 39 (38%) |
| **BMI (kg/m^2^)** | | | |
| Mean ± SD | 21.70±1.54 | 23.43±2.03 | 22.55±1.98 |
| ＜25 | 50 (96%) | 38 (76%) | 88 (86%) |
| ≥ 25 | 2 (4%) | 12 (24%) | 14 (14%) |
| **Medical history** | | | |
| Hypertension | 4 (8%) | 19 (38%) | 23 (23%) |
| Diabetes | 1 (2%) | 3 (6%) | 4 (4%) |
| **Alcohol** | | | |
| 0 g/week | 9 (17%) | 12 (24%) | 21 (21%) |
| 1-209 g/week | 33 (64%) | 15 (30%) | 48 (47%) |
| ≥ 210 g/week | 10 (19%) | 23 (46%) | 33 (32%) |
| **Tobacco** | | | |
| Never | 30 (58%) | 18 (36%) | 48 (47%) |
| Experienced | 14 (27%) | 23 (46%) | 37 (36%) |
| Current | 8 (15%) | 9 (18%) | 17 (17%) |

SD, Standard Deviation. BMI, Body Mass Index.

**Table S3** Clinicopathological characteristics of the CRC patients.

| **Characteristics** | | **N (%)** |
| --- | --- | --- |
| **TNM staging** | I | 8 (16%) |
|  | II | 14 (28%) |
|  | III | 26 (52%) |
|  | IV | 2 (4%) |
| **Primary origins** | Ascending colon | 19 (38%) |
|  | Transverse colon | 16 (32%) |
|  | Descending colon | 15 (30%) |
| **Pathological classification** | Adenocarcinoma (low differentiated) | 40 (80%) |
|  | Adenocarcinoma (moderately differentiated) | 8 (16%) |
|  | Mucinous adenocarcinoma | 2 (4%) |
| **Carcinoembryonic antigen** | Elevated | 47 (94%) |
|  | Normal | 3 (6%) |
| **Treatment** | Surgery | 16 (32%) |
|  | Chemotherapy | 0 (0%) |
|  | Surgery + neoadjuvant chemotherapy | 34 (68%) |

TNM, Tumor Node Metastasis.

**Table S4** Characteristics of the 14 samples selected for shotgun metagenomic sequencing.

| **Serial number** | **Gender** | **Place of birth** | **Age**  **(Year)** | **Height**  **(cm)** | **Weight**  **(kg)** | **BMI**  **(kg/m^2^)** | **TNM staging** | **Primary origins** | **Chronic inflammation** | **Gastrointestinal surgery** | **Antibiotic use within 2 months** | **Hypertension** | **Diabetes** | **Alcohol** | **Tobacco** |
| --- | --- | --- | --- | --- | --- | --- | --- | --- | --- | --- | --- | --- | --- | --- | --- |
| N16 | male | Shanghai | 49 | 174 | 58 | 19.15708812 |  |  | no | no | no | no | no | 1-209 g/week | current |
| N17 | female | Shanghai | 44 | 162 | 55 | 20.95717116 |  |  | no | no | no | no | no | none | never |
| N19 | male | Shanghai | 46 | 169 | 55 | 19.25702882 |  |  | no | no | no | no | no | none | never |
| N36 | male | Shanghai | 66 | 170 | 60 | 20.76124567 |  |  | no | no | no | no | no | 1-209 g/week | current |
| N38 | female | Shanghai | 63 | 158 | 55 | 22.03172568 |  |  | no | no | no | no | no | none | never |
| N51 | male | Shanghai | 58 | 168 | 56 | 19.84126984 |  |  | no | no | no | no | no | none | experienced |
| P3 | female | Shanghai | 63 | 155 | 60 | 24.97398543 | T2N1M0 | ascending colon | no | no | no | no | no | none | never |
| P6 | female | Zhejiang | 37 | 158 | 58 | 23.23345618 | T2N2M0 | transverse colon | no | no | no | no | no | 1-209 g/week | never |
| P11 | male | Jiangsu | 38 | 170 | 67 | 23.18339100 | T3N2M0 | ascending colon | no | no | no | no | no | ≥ 210 g/week | experienced |
| P13 | male | Shanghai | 58 | 170 | 67 | 23.18339100 | T2N1M0 | descending colon | no | no | no | no | no | ≥ 210 g/week | current |
| P16 | female | Shanghai | 68 | 166 | 63 | 22.86253448 | T2N1M0 | transverse colon | no | no | no | no | no | none | never |
| P24 | male | Shanghai | 64 | 169 | 63 | 22.05805119 | T2N2M0 | ascending colon | no | no | no | no | no | none | experienced |
| P42 | male | Shanghai | 47 | 183 | 73 | 21.79820239 | T2N2M0 | ascending colon | no | no | no | no | no | ≥ 210 g/week | never |
| P46 | male | Shanghai | 75 | 172 | 59 | 19.94321255 | T2N2M0 | ascending colon | no | no | no | no | no | 1-209 g/week | current |

**Table S5** Annotations of the ten most expressed orthologous genes and the corresponding KEGG pathways.

| **K00175: 2-oxoglutarate/2-oxoacid ferredoxin oxidoreductase subunit beta** | |
| --- | --- |
| map00010 | Glycolysis / Gluconeogenesis |
| map00020 | Citrate cycle (TCA cycle) |
| map00620 | Pyruvate metabolism |
| map00650 | Butanoate metabolism |
| map00720 | Carbon fixation pathways in prokaryotes |
| map01100 | Metabolic pathways |
| map01110 | Biosynthesis of secondary metabolites |
| map01120 | Microbial metabolism in diverse environments |
| map01200 | Carbon metabolism |
| **K01190: beta-galactosidase** | |
| map00052 | Galactose metabolism |
| map00511 | Other glycan degradation |
| map00600 | Sphingolipid metabolism |
| map01100 | Metabolic pathways |
| **K00382: dihydrolipoamide dehydrogenase** | |
| map00010 | Glycolysis / Gluconeogenesis |
| map00020 | Citrate cycle (TCA cycle) |
| map00260 | Glycine, serine and threonine metabolism |
| map00280 | Valine, leucine and isoleucine degradation |
| map00310 | Lysine degradation |
| map00380 | Tryptophan metabolism |
| map00620 | Pyruvate metabolism |
| map00630 | Glyoxylate and dicarboxylate metabolism |
| map00640 | Propanoate metabolism |
| map01100 | Metabolic pathways |
| map01110 | Biosynthesis of secondary metabolites |
| map01120 | Microbial metabolism in diverse environments |
| map01200 | Carbon metabolism |
| map01240 | Biosynthesis of cofactors |
| **K05349: beta-glucosidase** | |
| map00460 | Cyanoamino acid metabolism |
| map00500 | Starch and sucrose metabolism |
| map00999 | Biosynthesis of various plant secondary metabolites |
| map01100 | Metabolic pathways |
| map01110 | Biosynthesis of secondary metabolites |
| **K00615: transketolase** | |
| map00030 | Pentose phosphate pathway |
| map00710 | Carbon fixation in photosynthetic organisms |
| map01051 | Biosynthesis of ansamycins |
| map01100 | Metabolic pathways |
| map01110 | Biosynthesis of secondary metabolites |
| map01120 | Microbial metabolism in diverse environments |
| map01200 | Carbon metabolism |
| map01230 | Biosynthesis of amino acids |
| **K01897: long-chain acyl-CoA synthetase** | |
| map00061 | Fatty acid biosynthesis |
| map00071 | Fatty acid degradation |
| map01100 | Metabolic pathways |
| map01212 | Fatty acid metabolism |
| map02024 | Quorum sensing |
| map03320 | PPAR signaling pathway |
| map04146 | Peroxisome |
| map04216 | Ferroptosis |
| map04714 | Thermogenesis |
| map04920 | Adipocytokine signaling pathway |
| **K01624: fructose-bisphosphate aldolase** | |
| map00010 | Glycolysis / Gluconeogenesis |
| map00030 | Pentose phosphate pathway |
| map00051 | Fructose and mannose metabolism |
| map00680 | Methane metabolism |
| map00710 | Carbon fixation in photosynthetic organisms |
| map01100 | Metabolic pathways |
| map01110 | Biosynthesis of secondary metabolites |
| map01120 | Microbial metabolism in diverse environments |
| map01200 | Carbon metabolism |
| map01230 | Biosynthesis of amino acids |
| **K12373: hexosaminidase** | |
| map00511 | Other glycan degradation |
| map00513 | Various types of N-glycan biosynthesis |
| map00520 | Amino sugar and nucleotide sugar metabolism |
| map00531 | Glycosaminoglycan degradation |
| map00600 | Sphingolipid metabolism |
| map00603 | Glycosphingolipid biosynthesis - globo and isoglobo series |
| map00604 | Glycosphingolipid biosynthesis - ganglio series |
| map01100 | Metabolic pathways |
| map04142 | Lysosome |
| **K01915: glutamine synthetase** | |
| map00220 | Arginine biosynthesis |
| map00250 | Alanine, aspartate and glutamate metabolism |
| map00630 | Glyoxylate and dicarboxylate metabolism |
| map00910 | Nitrogen metabolism |
| map01100 | Metabolic pathways |
| map01120 | Microbial metabolism in diverse environments |
| map01230 | Biosynthesis of amino acids |
| map02020 | Two-component system |
| map04217 | Necroptosis |
| map04724 | Glutamatergic synapse |
| map04727 | GABAergic synapse |
| **K00850: 6-phosphofructokinase 1** | |
| map00010 | Glycolysis / Gluconeogenesis |
| map00030 | Pentose phosphate pathway |
| map00051 | Fructose and mannose metabolism |
| map00052 | Galactose metabolism |
| map00680 | Methane metabolism |
| map01100 | Metabolic pathways |
| map01110 | Biosynthesis of secondary metabolites |
| map01120 | Microbial metabolism in diverse environments |
| map01200 | Carbon metabolism |
| map01230 | Biosynthesis of amino acids |
| map03018 | RNA degradation |
| map04066 | HIF-1 signaling pathway |
| map04152 | AMPK signaling pathway |
| map04919 | Thyroid hormone signaling pathway |
| map04922 | Glucagon signaling pathway |
| map05230 | Central carbon metabolism in cancer |

**Table S6** Expression changes of Fusobacterium's 100 orthologous genes in CRC patients.

| **Abundance**  **ranking** | **Orthologous**  **gene** | **log10 (Fold**  **Change)** | **Abundance**  **ranking** | **Orthologous**  **gene** | **log10 (Fold**  **Change)** |
| --- | --- | --- | --- | --- | --- |
| 001 | K01588 | 0.344399741 | 051 | K01945 | 1.267709188 |
| 002 | K03551 | 0.333860172 | 052 | K02994 | 0.330784014 |
| 003 | K01740 | 1.153619893 | 053 | K00133 | 1.324493581 |
| 004 | K02996 | 0.316155696 | 054 | K00789 | 0.53340677 |
| 005 | K02110 | 0.386740659 | 055 | K00262 | 1.256216941 |
| 006 | K01915 | 0.537441563 | 056 | K04567 | 0.252831024 |
| 007 | K03648 | 0.613337168 | 057 | K03555 | 1.290910803 |
| 008 | K02961 | 0.358261349 | 058 | K02878 | 0.317824286 |
| 009 | K17828 | 0.188941232 | 059 | K02867 | 0.26858067 |
| 010 | K21636 | 0.779403766 | 060 | K01873 | 0.293379874 |
| 011 | K02965 | 0.383002496 | 061 | K01951 | 0.3431541 |
| 012 | K02874 | 0.44405201 | 062 | K01755 | 1.227900908 |
| 013 | K00602 | 0.474350814 | 063 | K01756 | 0.401563757 |
| 014 | K02909 | 0.548928575 | 064 | K00962 | 0.281188929 |
| 015 | K02946 | 0.324985259 | 065 | K02871 | 0.387331637 |
| 016 | K03701 | 0.396944622 | 066 | K01689 | 0.299735878 |
| 017 | K02954 | 0.33411695 | 067 | K02906 | 0.290922397 |
| 018 | K01955 | 0.391913747 | 068 | K01591 | 0.274129142 |
| 019 | K01883 | 0.333521725 | 069 | K02879 | 0.447861745 |
| 020 | K00850 | 0.329327626 | 070 | K02967 | 0.387319672 |
| 021 | K02899 | 0.557004158 | 071 | K01889 | 0.212477891 |
| 022 | K01867 | 0.259080272 | 072 | K00939 | 0.272241738 |
| 023 | K03553 | 0.279122446 | 073 | K01704 | 1.245545095 |
| 024 | K01872 | 0.713570015 | 074 | K01358 | 0.279613634 |
| 025 | K01687 | 1.417095435 | 075 | K01810 | 0.328412948 |
| 026 | K01790 | 1.861215801 | 076 | K04077 | 0.267264693 |
| 027 | K00790 | 0.295901463 | 077 | K02876 | 0.366411973 |
| 028 | K02935 | 0.30120008 | 078 | K01893 | 0.129711891 |
| 029 | K01923 | 0.453797303 | 079 | K02886 | 0.367809944 |
| 030 | K02950 | 0.194379772 | 080 | K02890 | 0.278821249 |
| 031 | K02952 | 0.342064984 | 081 | K01870 | 0.495764473 |
| 032 | K02902 | 1.122219248 | 082 | K02992 | 0.31121464 |
| 033 | K01874 | 0.553077451 | 083 | K03070 | 0.369179473 |
| 034 | K00615 | 0.352122728 | 084 | K02563 | 1.145349459 |
| 035 | K02988 | 0.341641082 | 085 | K01956 | 0.377053535 |
| 036 | K00625 | 0.287809547 | 086 | K00927 | 0.367288399 |
| 037 | K00973 | 1.137812239 | 087 | K03544 | 0.38474113 |
| 038 | K02881 | 0.359325793 | 088 | K00656 | 0.425340266 |
| 039 | K01738 | 1.274857141 | 089 | K00088 | 0.270210159 |
| 040 | K02358 | -0.121904145 | 090 | K02926 | 0.285406592 |
| 041 | K04043 | 0.341088369 | 091 | K01868 | 0.350341051 |
| 042 | K03695 | 0.366914229 | 092 | K00826 | 1.34448446 |
| 043 | K08303 | 0.57769394 | 093 | K18682 | 0.340239236 |
| 044 | K01933 | 0.355067343 | 094 | K03046 | 0.3512177 |
| 045 | K02112 | 0.299610215 | 095 | K00645 | 0.325592054 |
| 046 | K02911 | 0.113992039 | 096 | K01624 | 0.691299513 |
| 047 | K02111 | 0.370281894 | 097 | K00134 | 0.355301601 |
| 048 | K02948 | 0.402882127 | 098 | K01142 | 1.177272712 |
| 049 | K03086 | 0.555734749 | 099 | K01875 | 0.24065568 |
| 050 | K01940 | 1.26016966 | 100 | K02931 | 0.270137328 |
